# Amino acid transporter LAT1 in tumor-associated vascular endothelium promotes angiogenesis by regulating cell proliferation and VEGF-A-dependent mTORC1 activation

**DOI:** 10.1186/s13046-020-01762-0

**Published:** 2020-11-30

**Authors:** Lili Quan, Ryuichi Ohgaki, Saori Hara, Suguru Okuda, Ling Wei, Hiroki Okanishi, Shushi Nagamori, Hitoshi Endou, Yoshikatsu Kanai

**Affiliations:** 1 Department of Bio-system Pharmacology, Graduate School of Medicine, Osaka University, 2-2 Yamadaoka, Suita, 565-0871 Osaka Japan; 2 Present address: School of Traditional Chinese Medicine, Guangdong Pharmaceutical University, Guangzhou, 510006 Guangdong China; 3 Department of Laboratory Medicine, The Jikei University School of Medicine, Minato-ku, 634-8521 Tokyo Japan; 4 J-Pharma Co., Ltd, Yokohama, 230-0046 Kanagawa Japan; 5 Integrated Frontier Research for Medical Science Division, Institute for Open and Transdisciplinary Research Initiatives, Osaka University, Suita, 565-0871 Osaka Japan

**Keywords:** Amino acid transporter, Tumor angiogenesis, Endothelial cell, VEGF-A, mTORC1

## Abstract

**Background:**

Tumor angiogenesis is regarded as a rational anti-cancer target. The efficacy and indications of anti-angiogenic therapies in clinical practice, however, are relatively limited. Therefore, there still exists a demand for revealing the distinct characteristics of tumor endothelium that is crucial for the pathological angiogenesis. L-type amino acid transporter 1 (LAT1) is well known to be highly and broadly upregulated in tumor cells to support their growth and proliferation. In this study, we aimed to establish the upregulation of LAT1 as a novel general characteristic of tumor-associated endothelial cells as well, and to explore the functional relevance in tumor angiogenesis.

**Methods:**

Expression of LAT1 in tumor-associated endothelial cells was immunohistologically investigated in human pancreatic ductal adenocarcinoma (PDA) and xenograft- and syngeneic mouse tumor models. The effects of pharmacological and genetic ablation of endothelial LAT1 were examined in aortic ring assay, Matrigel plug assay, and mouse tumor models. The effects of LAT1 inhibitors and gene knockdown on cell proliferation, regulation of translation, as well as on the VEGF-A-dependent angiogenic processes and intracellular signaling were investigated in in vitro by using human umbilical vein endothelial cells.

**Results:**

LAT1 was highly expressed in vascular endothelial cells of human PDA but not in normal pancreas. Similarly, high endothelial LAT1 expression was observed in mouse tumor models. The angiogenesis in ex/in vivo assays was suppressed by abrogating the function or expression of LAT1. Tumor growth in mice was significantly impaired through the inhibition of angiogenesis by targeting endothelial LAT1. LAT1-mediated amino acid transport was fundamental to support endothelial cell proliferation and translation initiation in vitro. Furthermore, LAT1 was required for the VEGF-A-dependent migration, invasion, tube formation, and activation of mTORC1, suggesting a novel cross-talk between pro-angiogenic signaling and nutrient-sensing in endothelial cells.

**Conclusions:**

These results demonstrate that the endothelial LAT1 is a novel key player in tumor angiogenesis, which regulates proliferation, translation, and pro-angiogenic VEGF-A signaling. This study furthermore indicates a new insight into the dual functioning of LAT1 in tumor progression both in tumor cells and stromal endothelium. Therapeutic inhibition of LAT1 may offer an ideal option to potentiate anti-angiogenic therapies.

**Supplementary Information:**

The online version contains supplementary material available at 10.1186/s13046-020-01762-0.

## Background

Therapeutic intervention in tumor angiogenesis is one of rational strategies for anti-cancer treatment. Various agents including neutralizing antibodies and decoy receptors for pro-angiogenic factors, as well as antibodies and inhibitors for the receptor tyrosine kinases (RTKs), have been developed to target angiogenic signaling pathways in endothelial cells. Their efficacy and indications in clinical practice are, however, relatively limited [1, 2]. The redundancy in pro-angiogenic growth factor signaling with compensatory functions is one of the mechanisms accounting for the insufficient responsiveness and resistance to anti-angiogenic therapy [1, 2]. It was reported that treatment of rectal cancer patients with bevacizumab, an anti-VEGF antibody, increased the PlGF in plasma [3]. FGF-2 and PlGF were increased in glioblastoma multiforme patients treated with cediranib, a pan-VEGF receptor tyrosine kinase inhibitor [4, 5]. Similar upregulation of pro-angiogenic factors was also observed in mouse models of pancreatic islet tumor treated with anti-VEGFR2 antibody, where the expression of Ang-1, Ephrin-A1, Ephrin-A2, FGF-1, and FGF-2 was increased [6, 7]. The resultant tumor growth suppression was only transient with modest prolongation of survival [6, 7]. These results clearly indicate that the inhibition of a specific pro-angiogenic signaling pathway per se in endothelial cells is not sufficient to control the aberrant angiogenic activity in tumor.

To improve the clinical benefits of anti-angiogenic therapy, it is fundamental to understand the molecular signature of tumor-associated endothelium involved in the pathological blood vessel formation. L-type amino acid transporter 1 (LAT1) forms heterodimeric complex with its ancillary protein 4F2hc, and preferentially transports most of the essential amino acids [8, 9]. LAT1 is known to be upregulated in a wide spectrum of primary tumors and metastatic lesions from over 20 tissue/organ origins [10–12]. Furthermore, correlations between the LAT1 expression with poor prognosis have been indicated in various tumors including, but not limited to, triple negative breast cancer [13], highly proliferative ER^+^ subtype of breast cancer [14], bladder cancer [15], lung adenocarcinoma [16], lung neuroendocrine tumor [17], pancreatic ductal adenocarcinoma [18, 19], and biliary tract cancer [20]. LAT1 in cancer cells has, thus, been recognized as an emerging molecular target for anti-tumor therapy. Several LAT1-selective inhibitors have been synthesized [21–23], including JPH203 that showed prominent anti-tumor effects in preclinical animal models [21, 24–27]. The first-in-human phase I clinical trial was recently conducted in patients with advanced solid tumors, and reported that JPH203 appeared to be well-tolerated and to provide promising activity against biliary tract cancer [28].

Besides its well-recognized function in tumor cells, a yet unclarified role of LAT1 in tumor biology has been its implication to endothelial cell functions in tumors. An elevated expression of LAT1 in tumor-associated microvasculatures was reported in N-butyl-N-(4-hydroxybutyl) nitrosamine-induced rat bladder carcinoma model [29]. A clinicopathological study on human glioma showed LAT1 expression in both vascular endothelial cells and tumor cells, demonstrating significant correlations of LAT1 expression with the pathological grade and the intratumoral microvessel density [30]. These observations prompted us to hypothesize that LAT1 mediates amino acid supply not only to tumor cells, but also to tumor-associated endothelial cells, thereby promoting cellular functions related to angiogenesis. Here, we demonstrate the LAT1 expression is upregulated in tumor-associated blood vessels but not in the blood vessels of normal tissues in general. Functional relevance of endothelial LAT1 in tumor angiogenesis was investigated, pursuing the possibility of obtaining anti-angiogenic effects by targeting endothelial LAT1.

## Methods

### Chemicals

2-Aminobicyclo[2.2.1]heptane-2-carboxylic acid (BCH) was purchased from SIGMA-Aldrich. JPH203 ((S)-2-amino-3-(4-((5-amino-2-phenylbenzo [d]oxazol-7-yl)methoxy)-3,5-dichlorophenyl) propanoic acid, CAS No.: 1037592–40-7) (2HCl salt; purity > 99%), JPH203 sulfobutylether-β-cyclodextrin (JPH203-SBECD), and sulfobutylether-β-cyclodextrin (SBECD, placebo) were provided by J-Pharma Co., Ltd. Vascular endothelial growth factor A-165 (VEGF-A) and fibroblast growth factor-2 (FGF-2) of human and mouse recombinant proteins were purchased from WAKO Pure Chemical. Rapamycin was purchased from LC laboratories.

### Antibody production

A GST-fused recombinant protein of mouse LAT1 *N*-terminal 53 amino acids was expressed in *E.coli* BL21(DE3), and purified by Glutathione Sepharose 4B (GE Healthcare) affinity column chromatography. For rabbit antibody production (anti-mLAT1(R) antibody), a New Zealand White rabbit was intramuscularly immunized with the purified recombinant protein (200 μg in Freund’s complete adjuvant for the initial injection, followed by three times injection of 200 μg in incomplete Freund’s adjuvant with 2-week intervals). For chicken antibody production (anti-mLAT1(C) antibody), a White Leghorn chicken was immunized with the purified recombinant protein (200 μg in Freund’s complete adjuvant for the initial injection, followed by four times injection of 100 μg in incomplete Freund’s adjuvant with 2-week intervals). One week after the final injection, antisera were collected, passed through a GST-coupled Affi-Gel 10 column (Bio-Rad) for absorption of anti-GST antibody, and then subjected to purification by antigen-coupled Affi-Gel 10 column chromatography.

Reactivity and specificity of affinity purified antibodies were confirmed as shown in Supplementary Figure 1. Human embryonic kidney HEK293T cells (CRL-3216, ATCC), human colorectal cancer HT-29 cells (HTB-38, ATCC), mouse melanoma B16-F10 cells (CRL-6475, ATCC), and human lung cancer A549 cells (JCRB0076, JCRB) were cultured in DMEM supplemented with 10% FBS, and 100 units/mL penicillin - 100 μg/mL streptomycin (Nacalai Tesque). HEK293T cells were transfected with plasmids encoding *C*-terminally HA-tagged mouse LAT1 (pcDNA3.1(+)-mLAT1-HA), and mouse 4F2hc (pcDNA3.1(+)-m4F2hc). Lipofectamine 2000 (Invitrogen) was used for transfection according to the manufacturer’s protocol. Cells were used for assays 2 days after the transfection. Crude membrane fraction from the cultured cells was prepared and analyzed by western blotting. Cells grown on collagen I-coated cover slips were fixed in methanol and used for immunofluorescence as described previously [31]. Primary antibodies used were: anti-HA (11867423001, SIGMA-Aldrich), anti-LAT1 (KE026, TransGenic), anti-4F2hc (sc-7094, Santa Cruz Biotechnology), anti-mLAT1(C), and anti-mLAT1(R). Secondary antibodies used were: Alexa Fluor 488-conjugated donkey anti-chicken IgY (703–545-155) for anti-mLAT1(C), and Cy3-conjugated goat anti-rat IgG (112–165-143) for anti-HA from Jackson ImmunoResearch; Alexa Fluor 488-conjugated donkey anti-rabbit IgG (A21206) for anti-mLAT1(R), Alexa Fluor 568-conjugated donkey anti-rabbit IgG (A10042) for anti-LAT1, and Fluor568-conjugated donkey anti-goat IgG (A11057) for anti-4F2hc from Molecular Probes.

### Human umbilical vein endothelial cells

Human umbilical vein endothelial cells (HUVECs, Corning) were maintained in EGM-2 medium (Lonza) containing 2% FBS and growth factors (VEGF-A, FGF-2, EGF, and IGF-1) at 37 °C with 5% CO_2_/95% air. Experiments were performed using cells with passage numbers less than 9.

### Effect of VEGF-A and FGF-2 stimulation on LAT1 expression in HUVECs

HUVECs were seeded in collagen-coated 6 cm dish (1.0 × 10^4^ cells/dish). Two days later, cells were starved for VEGF-A and FGF-2 for 6 h, and then stimulated with either VEGF-A or FGF-2 alone, or in combination (10 ng/mL each). Total RNA and cell lysate were prepared and subjected to real-time PCR and western blotting, respectively.

### Gene knockdown by RNAi

HUVECs were seeded in collagen-coated 6 cm dish (0.7 ~ 1.0 × 10^4^ cells/dish). On the next day, Silencer Select siRNA for LAT1#1 (s15653), #2 (s15654), #3 (s15655), or Negative Control #2 (Ambion) was transfected using Lipofectamine RNAiMAX (Invitrogen). Cells were used for experiments 2 days after the transfection.

### Wound healing assay

HUVECs were seeded at 2.8 × 10^4^ cells in 70 μL of EGM-2 medium/well in 2-well silicone culture insert (ibidi GmbH) settled in 24-well plate, and incubated for 18 h. After starvation for serum and growth factors in EBM-2B medium (EBM-2 medium supplemented with 0.1% BSA) for 6 h, cell migration was initiated by removing the inserts and adding 1 mL/well of EBM-2B medium containing 10 ng/mL VEGF-A. DIC images were acquired immediately after removing the inserts to locate the initial edges of cell-free gaps. Cells were incubated for 12 h for migration, fixed by 4% paraformaldehyde (PFA), stained with crystal violet, and subjected to image acquisition. Bright field images were acquired using an inverted microscope (DMi1, Leica Microsystems). The number of migrated cells were counted by using Cell Counter plugin for ImageJ software (NIH).

### Invasion assay

HUVECs starved for serum and growth factors were seeded at 5 × 10^4^ cells in 250 μL of EBM-2B in the upper chamber of BioCoat Angiogenesis system: Endothelial Cell Invasion (Corning). Lower chamber was filled with 750 μL of EBM-2B medium containing 10 ng/mL VEGF-A. Cells were incubated for 12 h for invasion, stained by 0.5 μM Calcein-AM in HBSS for 1 h, and subjected to image acquisition from the bottom of chamber by a bright-field/fluorescence microscope (BZ-9000, Keyence). Area covered with invaded cells were calculated from binarized images by using ImageJ software.

### Tube formation assay

HUVECs starved for serum and growth factors were seeded at 1.0 × 10^4^ cells/well in 96-well plate coated with 50 μL/well of growth factor-reduced Matrigel. In each well, 100 μL of EBM-2B medium containing VEGF (10 ng/mL) was added. After incubation for 8 h, cells were stained with 3 μM Calcein-AM at 37 °C for 20 min, and subjected to image acquisition using a fluorescent microscopy (EVOS FL, Thermo Fisher Scientific). Total branching length was quantified by ImageJ software with Angiogenesis Analyzer plugin (http://image.bio.methods.free.fr/ImageJ/?Angiogenesis-Analyzer-for-ImageJ).

### Cell proliferation assay

HUVECs were seeded in collagen coated 96-well plates (1.0 × 10^3^ cells/well) in EGM-2 medium. BCH or JPH203 was added on the next day (Day 0). For LAT1 knockdown, cells were seeded at 48 h after siRNA transfection (Day 0). Cell proliferation was measured every 24 h for 3 days by CCK-8 kit (Dojindo).

### Aortic ring assay

Aortic ring assay was performed as described previously [32]. Serum-starved aortic rings from C57BL/6 J female mice were embedded in growth factor-reduced Matrigel, and cultured in the presence of 2.5% fetal bovine serum and 30 ng/mL VEGF. When indicated, BCH or JPH203 dihydrochloride was added into the medium. Doxycycline (DOX, 100 ng/mL) was added into the medium throughout the assays using DOX-inducible conditional LAT1-knockout mice. The numbers of sprouting microvessels were manually counted at 5 days after embedding.

### Matrigel plug assay

C57BL/6 J female mice of 8 ~ 10-week-old were subcutaneously injected at the inguinal region with 500 μL of high-concentration growth factor-reduced Matrigel (Corning) containing heparin (13 units) with or without 0.4 μg VEGF-A, 1.2 μg FGF-2, and BCH or JPH203 dihydrochloride. Ten days later, FITC-Dextran (2,000,000 MW, Invitrogen, 5 mg/mL saline, 100 μL/animal) was intravenously injected 20 min before the collection of Matrigel plugs. For spectrofluorometry quantification, plugs were lysed in DIVAA CellSperse (Trevigen). After spinning down debris, the fluorescence were measured by SH-9000Lab spectrofluorometer (excitation: 480 nm, emission: 520 nm. Corona Electric). Plugs without VEGF-A and FGF-2 were used for background subtraction of fluorescence.

### Construction of animal tumor models and quantification of blood vessels

Human pancreatic cancer MIA PaCa-2 cells (JCRB0070, JCRB) and lung cancer H520 cells (HTB-182, ATCC) were grown in DMEM (SIGMA-Aldrich) supplemented with 10% FBS (Gibco), and 100 units/mL penicillin - 100 μg/mL streptomycin (Nacalai Tesque). Before inoculation, cells were suspended in filtrated PBS, and mixed with growth factor-reduced Matrigel in a 1:1 volume ratio to give a final concentration of 2.5 × 10^7^ cells/mL. The cell suspension was subcutaneously injected into the lower flank of 6-week-old BALB/c-nu/nu female mice (5.0 × 10^6^ cells, 0.2 mL/animal). When indicated, the size of tumor was measured by caliper to calculate volumes using the formula: Tumor volume (mm^3^) = (length × width^2^)/2, where length and width are the longest and shortest dimensions of the tumor, respectively. Seven days later, when the tumor volume reached to 100 ~ 250 mm^3^, mice were divided into two groups (*n* = 5 for each group), and treated everyday with either JPH203-SBECD in saline (25 mg/kg/day, i.v.) or equivalent amount of placebo control. After 14 days of consecutive injection, tumors were excised and subjected to immunofluorescence analysis against CD34. From the acquired immunofluorescence images, binary images were generated by manual thresholding and used for the quantification of blood vessel density by “Analyze Particles” plugins of ImageJ software. Images were acquired from at least five randomly selected fields on each section, and 10 sections were analyzed for each tumor (50 ~ 100 pictures per tumor). Averaged numbers of blood vessels per mm^2^ tissue area for each tumor were used for statistical analysis.

An orthotopic syngeneic tumor model was constructed by subcutaneous inoculation of B16-F10 mouse melanoma cells (CRL-6475, ATCC) into *Lat1*^fl/fl^/*Tek-Cre* or control *Lat1*^fl/fl^ mice. B16-F10 cell suspension in PBS were mixed with growth factor-reduced Matrigel in a 1:1 volume ratio to give a final concentration of 2.5 × 10^6^ cells/mL. The cell suspension was subcutaneously injected into the lower flank of 6- to 8-week-old mice (0.5 × 10^6^ cells, 0.2 mL/animal). Tumor volumes were calculated every day as described above. Ten days after the implantation, the tumors were collected for the quantification of blood vessel formation using paraffin sections. To label intratumoral blood vessels, 100 μL of FITC-Dextran (2,000,000 MW, Invitrogen) solution (5 mg/mL in saline) was intravenously injected 35 min before the collection of tumors. Entire sections were analyzed to quantify the blood vessel area. From the acquired fluorescence images of FITC-dextran, binary images were generated by manual thresholding and used for the quantification of blood vessel area by “Analyze Particles” plugins of ImageJ software. Eight sections were analyzed for each tumor to calculate averaged blood vessel areas (μm^2^/mm^2^ tissue area), and used for statistical analysis. Experiments were performed with *n* = 4 for each group (*Lat1*^fl/fl^/*Tek-Cre* mice and control *Lat1*^fl/fl^ mice).

### Transgenic mice

*Lat1*^fl^ mice harboring floxed *Lat1* gene for conditional knockout were generated by Unitech Co., Ltd. Targeting construct was designed to excise exon 3 of *Lat1* gene (Supplementary Figure 2). A 1.2 kb-genomic region containing exon 3 was replaced by the corresponding genomic sequence flanked with a pair of loxP sequences. An FRT site-flanked neomycin resistance gene cassette was also inserted into the downstream of exon 3. Long and short arms (5.4 kb and 2.3 kb, respectively) were added for homologous recombination. All the genomic sequences were amplified from BAC clone RP23-46D12. A diphtheria toxin A-fragment (DTA) under thymidine kinase promoter was used for negative selection. The targeting construct was electroporated into mouse Bruce-4 ES cells derived from C57BL/6 J. After selection with 200 μg/ml of G418, successfully targeted ES clones were screened by PCR. Homologous recombination was further confirmed by Southern blot analysis using two external probes (5′- and 3′ probes against *SpeI*-digested genomic DNA) and an internal probe (Neo probe against *EcoRV*-digested genomic DNA). Positive ES clones were then injected into Balb/c blastocysts to obtain chimeric mice. Germ line transmission was established by crossing the chimeric mice with C57BL/6 J mice, and obtained heterozygous founder mice were further crossed with *CAG-FLP* mice expressing Flp-recombinase under the control of the CAG-promoter, to excise the FRT site-flanked neomycin resistance cassette. After confirming the removal of neomycin resistance gene cassette by PCR, the resultant *Lat1*^fl^ mice were maintained with C57BL/6 J genetic background.

For conditional knockout of *Lat1* gene, *Lat1*^fl^ mice were crossed with following transgenic mice. *CAG-rtTA3* mice expressing reverse tetracycline-controlled transactivator 3 (rtTA3) under the control of CAG promoter (B6N.FVB (Cg)-Tg (CAG-rtTA3)4288Slowe/J) [33], and *TetO-Cre* mice harboring Cre recombinase under the control of tetracycline-responsive promoter element (B6.Cg-Tg (tetO-cre)1Jaw/J) [34] were obtained from Jackson Laboratory. *Tek-Cre* mice expressing Cre recombinase gene under endothelial cell specific Tek promoter/enhancer (B6.Cg-Tg (Tek-cre)1Ywa) [35] were from RIKEN BioResource Center. To avoid non-cell-specific deletion of floxed alleles by the female germ line activation of Tek promoter [36], *Tek-Cre* positive female mice were not used for mating. Genotyping PCR was routinely performed by KOD One PCR Master Mix (TOYOBO) using genomic DNA extracted from tail tips. *CAG-rtTA3*, *TetO-Cre*, and *Tek-Cre* transgenes were analyzed by protocols provided by their resources. Wild type allele and floxed allele of *Lat1* gene were distinguished by following primers: Fw (5′-TATAGAGAGAGACTTGGGATGAAGC-3′), Rv (5′-CAGCACACTGATTGTGACAAAGG-3′). Floxed allele and knockout allele of *Lat1* gene were distinguished by following primers: Fw (5′-GTTTCCAGTCTGGCATCTTTAAGTAG-3′), Rv (5′-CCCTGTGCTCAGACAGAAATGAGA-3′).

### Amino acid transport measurement and western blotting in *X. laevis* oocytes

Experiments using *X. laevis* oocytes shown in Supplementary Figure 3 were conducted basically as described previously [37]. Defolliculated oocytes were injected with in vitro transcribed polyadenylated cRNA (25 ng per oocyte). Equimolar of 4F2hc cRNA was co-injected for the co-expression with LAT1 or LAT1-Δex3. The oocytes were used for assays 2 days after injection. For transport measurement, oocytes were incubated at room temperature for 15 min with 500 μl of Na^+^-free uptake buffer (96 mM Choline-Cl, 2 mM KCl, 1.8 mM CaCl_2_, 1 mM MgCl_2_ and 5 mM HEPES [pH 7.5]) containing 100 μM of ^14^C-labeled l-leucine (l-[^14^C] Leu [3.3 Ci/mol, Moravek]). The radioactivity was determined by liquid scintillation counting. For western blotting using total membrane fractions of oocytes, following antibodies were used for the detection of LAT1 and 4F2hc: anti-mLAT1(R), anti-4F2hc (sc-7094, Santa Cruz Biotechnology), peroxidase-conjugated goat anti-rabbit IgG (111–035-003, Jackson ImmunoResearch), and peroxidase-conjugated mouse anti-goat IgG (205–035-108, Jackson ImmunoResearch).

### Immunostaining

Immunohistochemistry and immunofluorescence of tissue sections were performed as described previously [31]. Tissue blocks of pancreatic ductal adenocarcinoma (IDs: CU1372–35-35,006/13 T and CU1372–35-42,720/12 T) and of normal pancreas (IDs: CU2012/07 S12-33B and CU2009/02 X-40) were purchased from Cureline. Tissue microarray of pancreatic cancer (Array name: PA1001b) was purchased from US Biomax. Primary antibodies used are: anti-LAT1 (KE026, TransGenic), anti-mLAT1(R) (this study), anti-mLAT1(C) (this study), anti-CD34 (sc-18917, Santa Cruz Biotechnology), and anti-CD31 (sc-1506, Santa Cruz Biotechnology). For immunohistochemistry, sections treated with primary antibodies were further treated with biotinylated secondary antibody followed by incubation with avidin-biotin-peroxidase complex (VECTASTAIN ABC Elite Kit, Vector Laboratories, Inc.). Immunoreactive signals were developed by Peroxidase Stain DAB Kit (Nacalai Tesque). Nuclei were counterstained with Hematoxylin. For immunofluorescence, fluorescently labeled secondary antibodies used are: Alexa Fluor 488-conjugated goat anti-rabbit IgG (A11008) or Alexa Fluor 568-conjugated donkey anti-rabbit IgG (A10042) for anti-mLAT1(R), and Alexa Fluor 568-conjugated donkey anti-goat IgG (A11057) for anti-CD31, all of which were from Molecular Probes; Alexa Fluor 488-conjugated donkey anti-chicken IgY (703–545-155) for anti-mLAT1(C), and Cy3-conjugated goat anti-rat IgG (112–165-143) for anti-CD34 from Jackson ImmunoResearch.

Whole-mount immunofluorescence of aortic rings was performed as described previously [38] with minor modifications. Serum-starved aortic rings were embedded into Matrigel on 35 mm glass-bottom dish. After incubation for 3 days, aortic rings were fixed in 4% PFA at 4 °C overnight, washed twice in PBS, and permeabilized with 0.5% Triton X-100/PBS for 1 h. Blocking was performed for 2 h in PBS containing 0.5% Triton X-100 and 1% BSA at room temperature. Incubation with primary- [anti-mLAT1(C), and anti-Claudin5 (sc-28670, Santa Cruz Biotechnology)] and secondary antibodies [Alexa Fluor 488-conjugated donkey anti-chicken IgY, and Alexa Fluor 568-conjugated goat anti-rabbit IgG] were performed at 4 °C overnight, followed by washing with PBS for 30 min for three times. DAPI was used for nucleus staining. Stained samples were observed under an inverted confocal laser scanning microscope (FV-1000; Olympus).

### Real-time PCR

Total RNA from HUVECs and that from mouse aorta were extracted using Isogen II (Nippon Gene) and Agencourt RNAdvance Tissue Kit (Beckman Coulter), respectively. Quantitative real-time PCR was performed as described previously [31].

### Western blotting

Total cell lysates of HUVECs were prepared as described previously [39]. Crude membrane fractions were prepared as previously [40], and solubilized on ice for 30 min with 1% NP-40. After mixing with Laemmli buffer, SDS-polyacrylamide gel electrophoresis and western blot analysis were performed [39]. The antibody-treated PVDF membrane was developed with ECL Prime Western Blotting Detection System and imaged by Amersham Imager 680 (GE Healthcare).

Primary antibodies used are as follows: anti-Na^+^/K^+^-ATPase α1 (sc-21712), anti-p70S6K (sc-230), anti-Akt (sc-1618), anti-Src (sc-8056), and anti-PLCγ (sc-7290) from Santa Cruz Biotechnology; anti-phospho-Thr389-p70S6K (9243), anti-S6 ribosomal protein (2217), anti-phospho-Ser235/Ser236-S6 ribosomal protein (4858), anti-eIF2α (eukaryotic initiation factor 2α subunit) (5324), anti-phospho-Ser51-eIF2α (3398), anti-phospho-Thr308-Akt (13038), anti-phospho-Ser473-Akt (4060), anti-Erk1/2 (4696), anti-phospho-Thr202/Tyr204-Erk1/2 (4370), anti-VEGFR2 (9698), anti-phospho-Tyr1175-VEGFR2 (2478), anti-p38 (8690), anti-phospho-Thr180/Tyr182-p38 (4511), anti-phospho-Tyr416-Src (6943), anti-FAK (3285), anti-phospho-Tyr397-FAK (8556), and anti-phospho-Ser1248-PLCγ (8713) from Cell Signaling Technology; anti-LAT1 (KE026) from TransGenic; anti-β-actin (66009–1-Ig) from Proteintech.

### Statistical analysis

Statistical analyses were performed with GraphPad Prism8 (GraphPad software) by unpaired two-tailed Student’s t-test for Figs. 2b, d, 3a, b, c, d, 4b, e, j, and 5c, one-way ANOVA followed by Tukey’s post-test for Figs. 5a, 6b, d, and f, and two-way ANOVA followed by Tukey’s post-test for Figs. 4a, g, 5b, e, and f. Differences were considered significant when *p*-values were < 0.05. * *p* < 0.05, ** *p* < 0.01, *** *p* < 0.001, **** *p* < 0.0001, ns, not significant. Data are shown as mean ± s.e.m. in Figs. 2, 3, 4, 5c, and 6, and mean ± s.d. in Fig. 5a, b, e, and f.

## Results

### LAT1 is expressed in tumor-associated endothelial cells

**Fig. 1:**
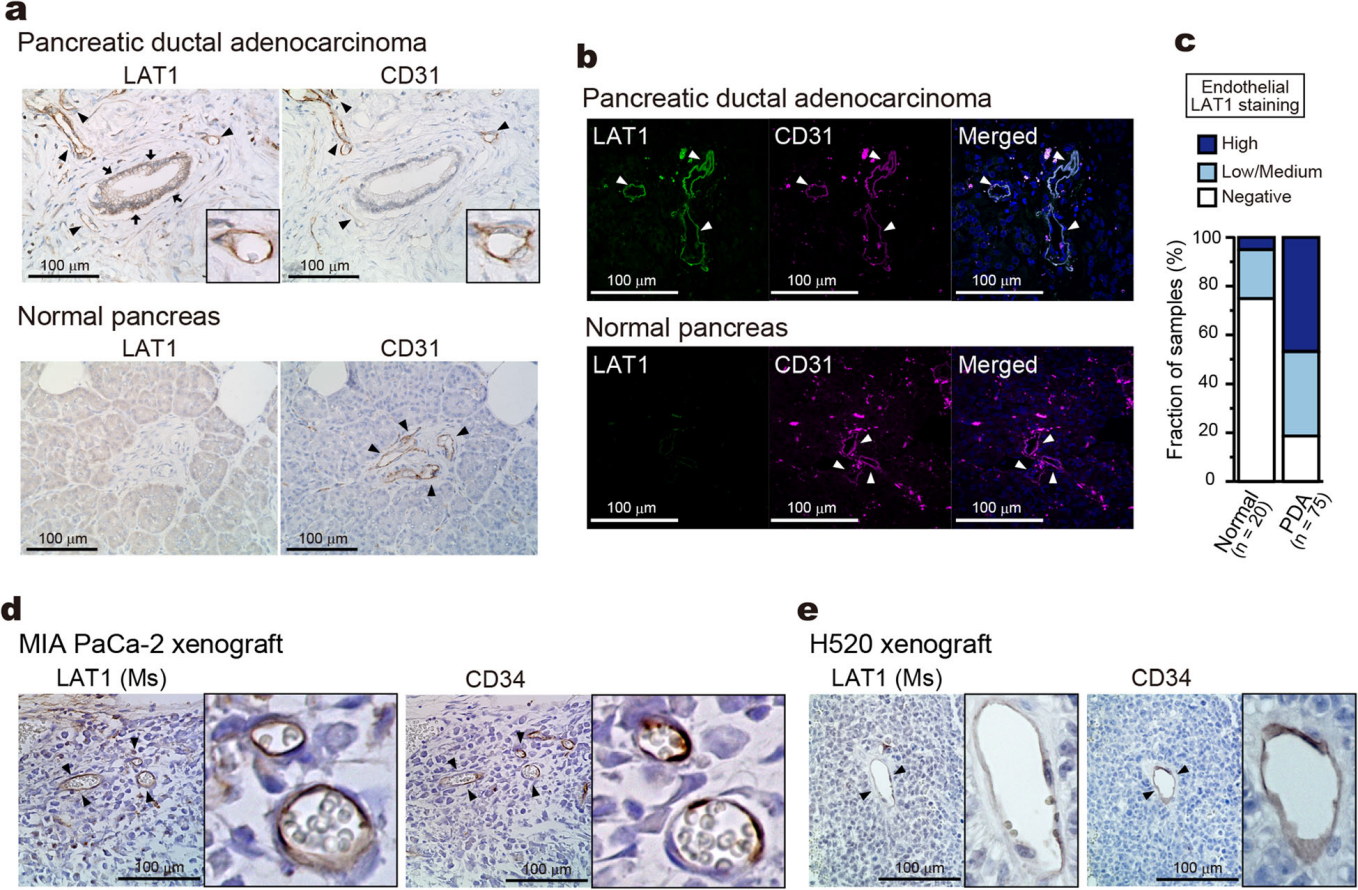
Expression of LAT1 in tumor-associated vascular endothelial cells. **a** Immunohistochemistry of LAT1 and CD31 in pancreatic ductal adenocarcinoma (PDA) and normal pancreas. Representative images from CU1372–35-35,006 (PDA) and CU2009/02 X-40 (normal pancreas) are shown. Arrows and arrowheads indicate tumor cells and endothelial cells, respectively. *Black squares*; enlarged images. **b** Immunofluorescence of LAT1 (*green*) and CD31 (*purple*) in PDA and normal pancreas. Nuclei stained with DAPI *(blue*) are shown in the merged images. **c** Endothelial LAT1 expression in tissue microarray containing PDA and normal pancreas. Tissues spots were classified depending on the LAT1 staining intensity in endothelial cells: High, Low/Medium, and Negative. Data shown are the percentage of each group. **d** and **e** Immunohistochemistry of mouse LAT1 and CD34 in paraffin sections of MIA PaCa-2 (**d**) and H520 (**e**) xenograft tumors. Arrowheads indicate endothelial cells. *Black squares*; enlarged images. Mouse LAT1 was visualized with mouse-specific mLAT1(**c**) antibody (*LAT1(Ms)*)

Upregulation of LAT1 has been reported in cancers of various tissue origins including pancreatic ductal adenocarcinoma (PDA) [18, 19]. Consistently, a high expression of LAT1 was detected in cancer cells of PDA tissue in our immunohistochemistry (Fig. 1a). Intriguingly, we also noticed a significant expression of LAT1 in the stromal cells that are positive for an endothelial cell marker CD31 (Fig. 1a). Endothelial cells in normal pancreatic tissue were, in contrast, mostly negative for LAT1 staining. The colocalization of LAT1 and CD31 in the tumor-associated endothelial cells of PDA tissue was further demonstrated by immunofluorescence (Fig. 1b). This observation was confirmed in a larger number of samples on tissue microarray by immunohistochemistry (Fig. 1c). Only a minor fraction (25.0%) of normal pancreatic tissues exhibited positive LAT1 staining in endothelial cells: 4 showed low-to-moderate, and 1 showed strong staining among 20 analyzed tissue spots. In contrast, a majority of PDA tissues (81.4%) exhibited positive LAT1 expression in endothelial cells: 26 showed low-to-moderate, and 35 showed strong staining among 75 analyzed tissue spots.

The expression of LAT1 in tumor-associated blood vessels was further examined in human cancer-cell xenograft tumor models in athymic nude mice. To detect mouse LAT1 in blood vessels surrounded by cancer cells highly expressing human LAT1, we generated mouse LAT1-specific antibodies (Supplementary Figure 1). Using the obtained antibody, mouse LAT1 was detected in CD34-positive endothelial cells in the tumors of pancreatic cancer MIA PaCa-2 cells (Fig. 1d) and of non-small cell lung cancer H520 cells (Fig. 1e). In contrast, no clear LAT1 staining was detected in the blood vessels of normal tissues except brain capillaries, where the expression of LAT1 has been reported previously [41, 42] (Supplementary Figure 4). The expression of LAT1 in the endothelial cells of tumor-associated blood vessels was, thus, recapitulated in the xenograft tumor models of distinct tissue origins.

### Endothelial LAT1 contributes to angiogenesis in ex- and in vivo assays

**Fig. 2:**
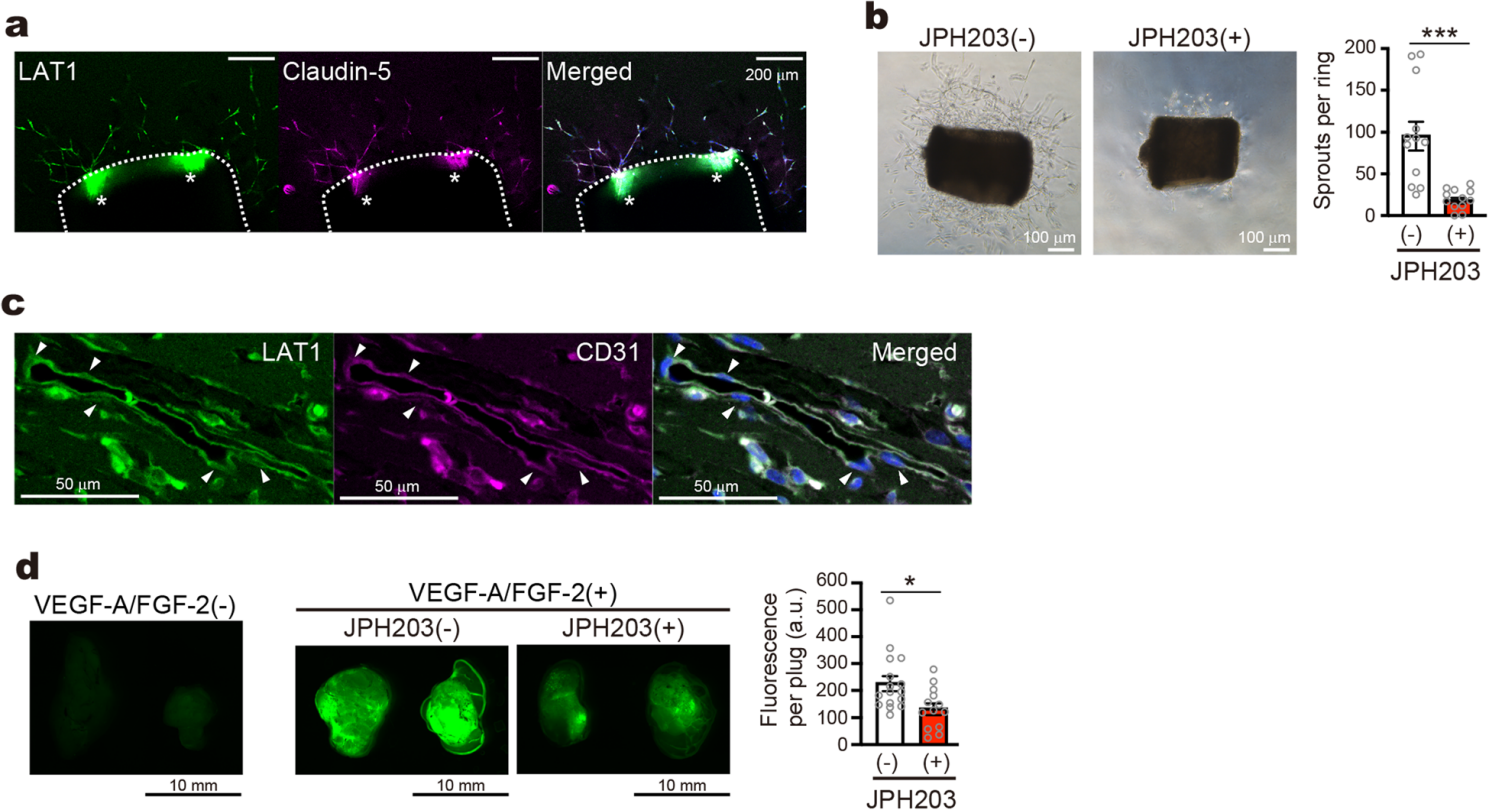
Effects of LAT1 inhibition in aortic ring assay and in Matrigel plug assay. **a** Whole-mount immunofluorescence of LAT1 and Claudin-5 in aortic rings. *Dashed lines*; edge of aortic rings. *Asterisks*; autofluorescence from aortic rings. **b** Aortic ring assay performed in the presence or the absence of 50 μM JPH203. *Bar graph*; quantification of endothelial sprouts. **c** Immunofluorescence of LAT1 and CD31 in Matrigel plug frozen section. **d** Fluorescent images of isolated Matrigel plugs implanted with or without 50 μM JPH203. *Bar graph*; quantification of FITC fluorescence. **a**, **c** LAT1 was stained with mLAT1 (**c**) antibody. Nuclei were stained with DAPI (*blue*, in merged image). **b**, *n* = 12; **d**, *n* = 16 for non-treated control (−), *n* = 13 for JPH203

The results above prompted us to investigate the functional relevance of endothelial LAT1 in angiogenesis. We first performed aortic ring assay, in which endothelial microvessel-like sprouts grew out from the slice of aorta in the Matrigel. Expression of LAT1 in the endothelial sprouts were confirmed by whole-mount immunofluorescence with an endothelial marker Claudin-5 (Fig. 2a). To examine the effects of pharmacological inhibition of LAT1, Matrigel-embedded aortic rings were cultured in the presence of JPH203 or BCH. Both of the compounds suppressed the outgrowth of endothelial sprouts to 10 ~ 20% of control (Fig. 2b and Supplementary Figure 5A).

We then performed Matrigel plug assay, in which Matrigel was subcutaneously injected into mice and analyzed for blood vessel formation. Immunofluorescence revealed that LAT1 is expressed in the CD31-positive endothelial cells within the Matrigel plugs (Fig. 2c). Pro-angiogenic growth factors, VEGF-A and FGF-2, mixed with Matrigel induced vascularization, as demonstrated by the higher fluorescence of intravenously injected FITC-dextran. LAT1 inhibition by BCH and JPH203 mixed in Matrigel reduced the fluorescence of the plugs, indicating a decreased angiogenesis (Fig. 2d and Supplementary Figure 5B).

To obtain further evidence for the roles of endothelial LAT1 in angiogenesis, we generated conditional knockout mice harboring exon 3-floxed *Lat1* allele (Supplementary Figure 2). The deletion of exon 3 in mouse *Lat1* gene results in an early frameshift and creates a premature stop codon. As a consequence, an *N*-terminal fragment of LAT1 composed of 227 amino acids (containing TM1-TM5) followed by two unrelated amino acids (−Thr-Ile) would be potentially expressed. The corresponding LAT1 fragment (LAT1-Δex3) co-expressed with 4F2hc did not exhibit any l-[^14^C] leucine transport function in *Xenopus* oocytes (Supplementary Figure 3A). The protein expression of LAT1-Δex3 in oocyte membrane fraction was markedly lower than that of wild type LAT1. LAT1-Δex3 did not form a heterodimer with 4F2hc (Supplementary Figure 3B).

**Fig. 3:**
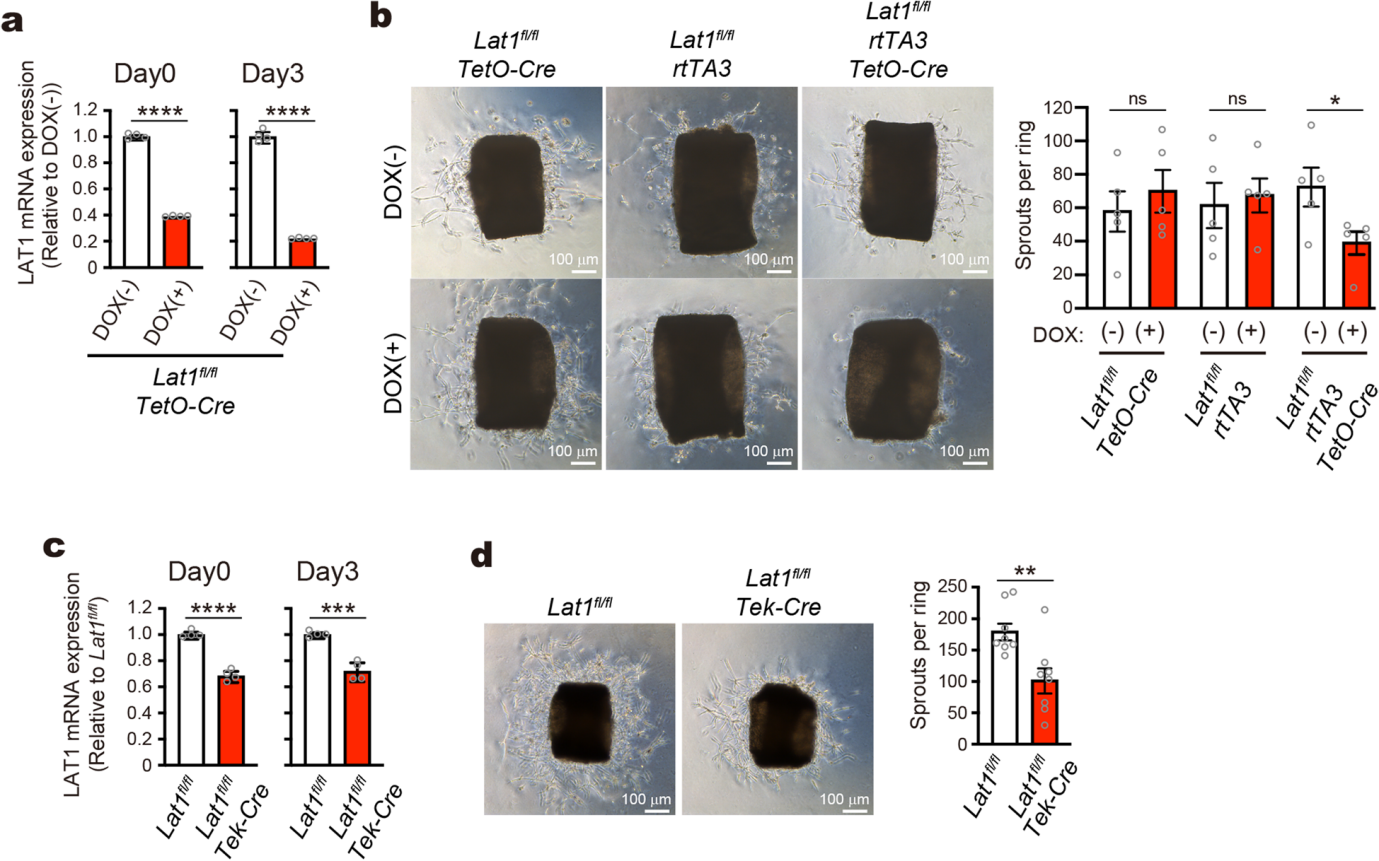
Suppression of endothelial sprouting in aortic ring assay by genetic ablation of LAT1. **a** LAT1 mRNA expression in Matrigel-embedded aortic rings of *Lat1*^fl/fl^/*rtTA3*/*TetO-Cre* mice analyzed on the day of embedding (Day 0) and three days later (Day 3), with or without DOX-treatment. **b** Aortic ring assay using *Lat1*^fl/fl^/*rtTA3*/*TetO-Cre* mice and control littermates (*Lat1*^fl/fl^/*rtTA3* and *Lat1*^fl/fl^/*TetO-Cre*). **c** LAT1 mRNA expression in aortic rings of *Lat1*^fl/fl^*/Tek-Cre* mice and control littermates (*Lat1*^fl/fl^) analyzed on Day 0 and Day 3. **d** Aortic ring assay using *Lat1*^fl/fl^*/Tek-Cre* and *Lat1*^fl/fl^ mice. **b**, **d** *Bar graphs*; quantification of endothelial sprouts. **a**, **c**, *n* = 4; **b**, *n* = 5; **d**, *n* = 8

The doxycycline (Dox)-inducible conditional knockout *Lat1*^fl/fl^/*rtTA3*/*TetO-Cre* mice were generated from the *Lat1*^fl^ mice (Supplementary Figure 6A). In the aortic rings isolated from the *Lat1*^fl/fl^/*rtTA3*/*TetO-Cre* mice, DOX-treatment decreased the LAT1 mRNA expression to ~ 40% of the control without DOX-treatment on the day of embedding (Day 0, after overnight DOX-treatment with serum starvation), and to ~ 20% of the control 3 days after embedding (Day 3) (Fig. 3a). We found that the outgrowth of endothelial sprouts was suppressed by DOX-treatment in the aortic rings prepared from *Lat1*^fl/fl^/*rtTA3*/*TetO-Cre* mice, whereas not in those from control *Lat1*^fl/fl^/*rtTA3* and *Lat1*^fl/fl^*/TetO-Cre* mice (Fig. 3b). To exclude the contribution of non-endothelial cells, we used endothelial cell-specific knockout mice (Supplementary Figure 6B). Depletion of endothelial LAT1 protein in *Lat1*^fl/fl^/*Tek-Cre* mice was evidenced by the immunofluorescence using brain sections (Supplementary Figure 6C). LAT1 mRNA was decreased in the aortic rings of the *Lat1*^fl/fl^/*Tek-Cre* mice to ~ 70% of the control *Lat1*^fl/fl^ mice on Day 0 and Day 3 (Fig. 3c). The endothelial sprouting was significantly suppressed in the aortic rings of *Lat1*^fl/fl^/*Tek-Cre* mice, further supporting the contribution of endothelial LAT1 (Fig. 3d).

### Genetic and pharmacological inhibition of endothelial LAT1 suppress angiogenesis and tumor growth

**Fig. 4:**
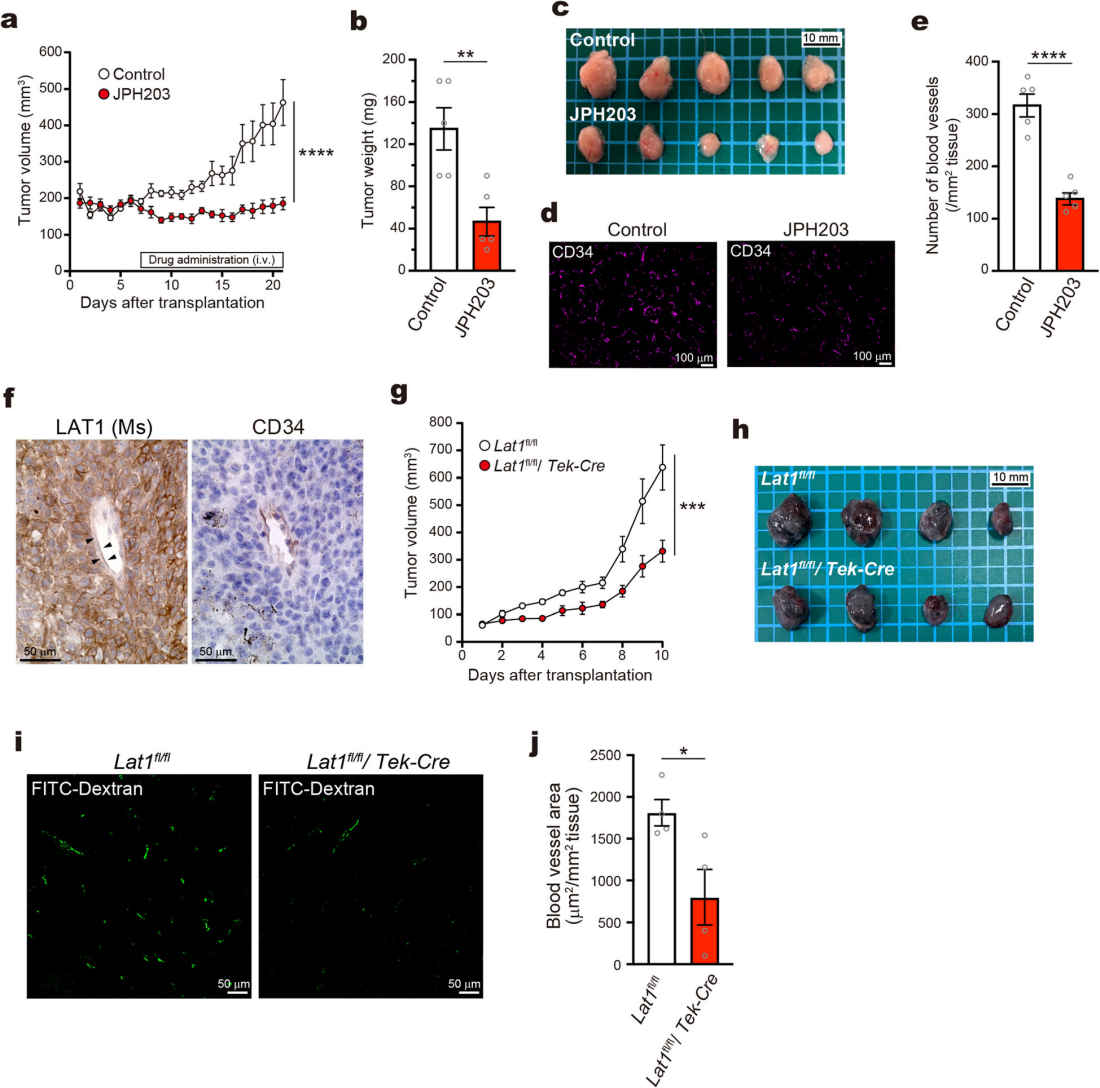
Anti-angiogenic effects of LAT1 inhibition and endothelial-specific LAT1 depletion in in vivo tumor models. **a** Growth of MIA PaCa-2 xenograft tumors in athymic nude mice. White bar indicates the period of daily drug administration. Control, placebo treatment; JPH203, 25 mg/kg/day i.v. (*n* = 5 each). **b** Tissue weight and **c** gross appearance of xenograft tumors collected on Day 21. **d** Immunofluorescence of CD34 in the tumor paraffin sections. **e** Quantification of the density of CD34-positive intratumoral blood vessels. **f** Immunohistochemistry of LAT1 and CD34 in the paraffin sections of orthotopic syngeneic tumor model of B16-F10 cells in C57BL/6 J mice. LAT1 was stained with mLAT1 (**c**) antibody (*LAT1(Ms)*). Arrowheads indicate endothelial staining of LAT1. **g** Growth of B16-F10 tumors in *Lat1*^*fl/fl*^ and *Lat1*^*fl/fl*^*/Tek-Cre* mice (*n* = 4 each). **h** Gross appearance of tumors collected on Day 10. **i** Intratumoral blood vessels visualized by intravenously injected FITC-Dextran. **j** Quantification of the blood vessel area on tumor sections

It has been demonstrated that LAT1 inhibitor JPH203 suppresses the xenograft tumor growth [21, 24–27]. In the present study, intravenous administration of JPH203 drastically suppressed the growth of MIA PaCa-2 xenograft tumors (Fig. 4a-c). Concomitantly, the intratumoral blood-vessel density was reduced in JPH203-treated tumors to ~ 45% of the placebo-treated control (Fig. 4d and e), which let us speculate that the reduction of tumor angiogenesis could contribute to the anti-tumor effects of JPH203.

We then examined whether the depletion of endothelial LAT1 suppresses tumor angiogenesis and, consequently, suppresses tumor growth. In the orthotopic syngeneic tumor model of B16-F10 mouse melanoma cells, expression of LAT1 was confirmed in tumor-associated endothelial cells as well as tumor cells (Fig. 4f). When the tumor was constructed in *Lat1*^fl/fl^/*Tek-Cre* mice, the growth was significantly suppressed compared to that in control mice (Fig. 4g and h). The analysis of the intratumoral blood vessel density revealed that the blood-vessel area in tumors was decreased in *Lat1*^fl/fl^/*Tek-Cre* mice to ~ 50% of that in the control mice (Fig. 4i and j). These results demonstrate that LAT1 in tumor-associated endothelial cells plays essential roles in tumor angiogenesis, and that the suppression of its function or expression could contribute to exert anti-tumor effects.

### LAT1 supports proliferation of endothelial cells via mTORC1- and GAAC pathways

**Fig. 5:**
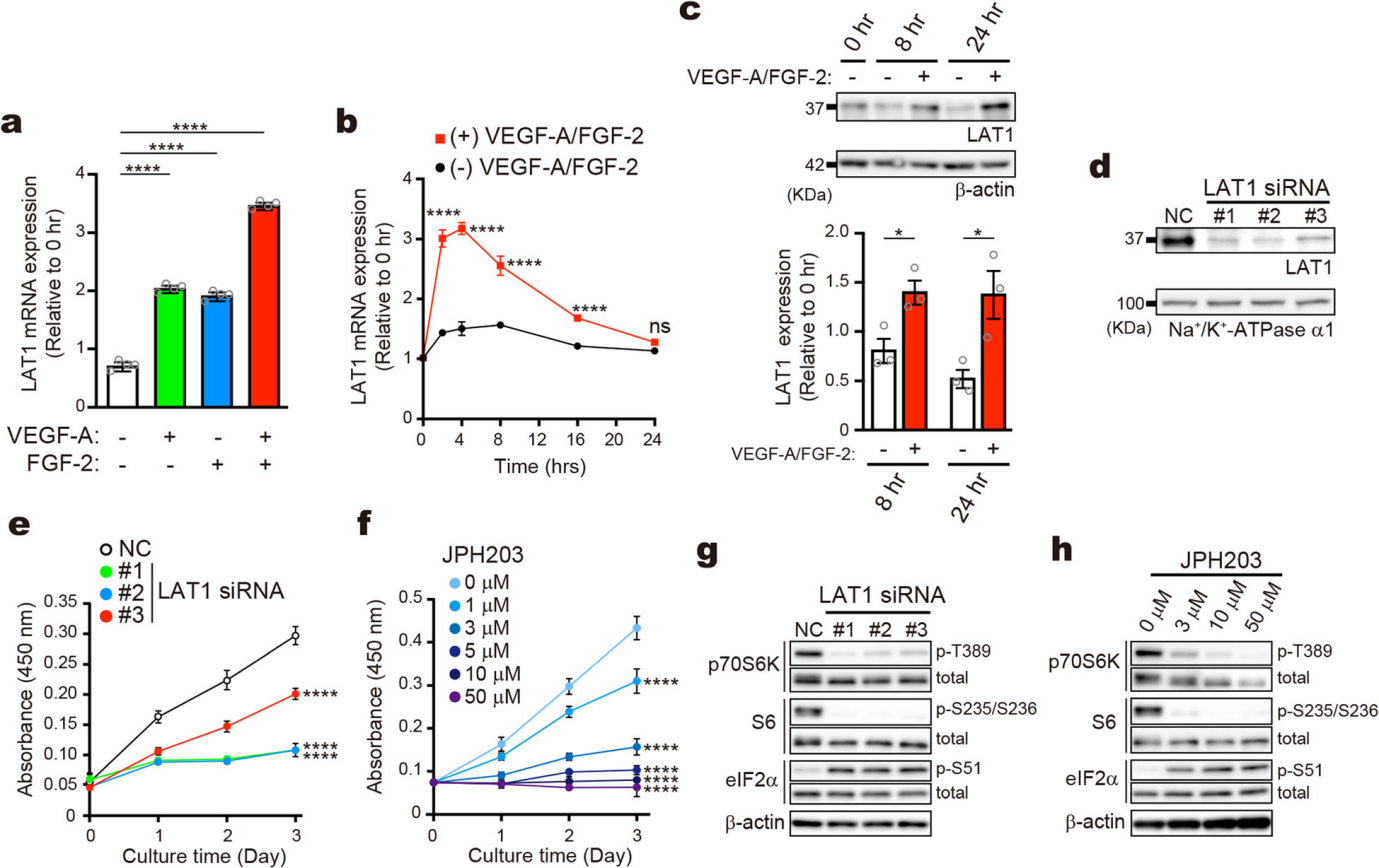
Endothelial LAT1 expression induced by pro-angiogenic factors and its contribution to proliferation and translation regulation. **a** and **b** LAT1 mRNA expression in HUVECs starved for VEGF-A and FGF-2 followed by stimulation for 2 h with VEGF-A or FGF-2 alone (10 ng/mL), or with their combination (**a**), or for 24 h with VEGF-A and FGF-2 (10 ng/mL each) (**b**). **c** LAT1 protein expression in HUVECs starved as in (**a** and **b**), then stimulated with VEGF-A and FGF-2 (10 ng/mL each). Cell lysates were analyzed by western blotting. *β-actin*; loading control. *Bar graph*; densitometric quantification of the band intensity. **d** LAT1 knockdown (KD) in HUVECs. Crude membrane fractions prepared 48 h after transfection of control (*NC*) or LAT1-targeting siRNAs (*LAT1 siRNA #1–3*) were examined by western blotting. *Na*^*+*^*/K*^*+*^*-ATPase α 1*; loading control. **e** and **f** Cell proliferation of LAT1 KD cells (**e**) and JPH203-treated cells (**f**). **g** and **h** Effects of LAT1 KD (**g**) and JPH203 (**h**) on mTORC1- and GAAC pathways. Cell lysates were prepared and analyzed 48 h after transfection of siRNAs (**g**), and after JPH203-treatment for 24 h (**h**). **a**, **b**, *n* = 4; **c**, *n* = 3; **e**, **f**, *n* = 8

Because LAT1 preferentially transports many essential amino acids, we investigated the importance of LAT1 in the endothelial cell proliferation using human umbilical vein endothelial cells (HUVECs). The culture medium of HUVECs is supplemented with major pro-angiogenic factors, VEGF-A and FGF-2. As shown in Fig. 5a, VEGF-A or FGF-2 alone, as well as their combination induced LAT1 mRNA expression in HUVECs. The combinational effect of VEGF-A and FGF-2 on LAT1 mRNA expression peaked at 2 ~ 4 h, and was sustained as long as 16 h (Fig. 5b). A consistent increase in the LAT1 protein amount was observed at 8 and 24 h after the stimulation with VEGF-A and FGF-2 (Fig. 5c). These results suggest that VEGF-A and FGF-2 could contribute to the induction of endothelial LAT1 expression under pro-angiogenic conditions.

The knockdown (KD) of LAT1 by siRNAs, that reduced the LAT1 protein amount to 15 ~ 25% of the control (Fig. 5d), impaired the proliferation of HUVECs (Fig. 5e). Similarly, LAT1 inhibition by JPH203 or BCH suppressed the proliferation of HUVECs in concentration dependent manners (Fig. 5f and Supplementary Figure 5C). These results showed that LAT1 plays a crucial role for the endothelial cell proliferation.

Amino acids are essential signaling molecules to activate a serine/threonine kinase complex mTORC1 (mechanistic target of rapamycin complex 1) that integrates nutrient- and growth factor signaling to support cell growth and proliferation [43]. Most well-characterized downstream effectors of mTORC1 include ribosomal protein S6 kinase p70S6K, a regulator of translation initiation. The accumulation of uncharged tRNAs under amino acid deficiency also activates the other signaling pathway, known as general amino acid control (GAAC) pathway [44, 45]. Uncharged tRNAs activate general control nonderepressible 2 (Gcn2) kinase and induce phosphorylation of eIF2α, which triggers a global down-regulation of translation by inhibiting the recruitment of initiator methionyl-tRNA to ribosome. As shown in Fig. 5g and h, LAT1 KD as well as LAT1 inhibition by JPH203 in HUVECs markedly reduced the phosphorylation of p70S6K and its substrate ribosomal protein S6. The phosphorylation of eIF2α was also increased, indicating the activation of GAAC pathway by amino acid deficiency. Collectively, these results indicate that LAT1-mediated amino acid transport in HUVECs is an essential prerequisite to activate translation initiation. The inhibition of endothelial LAT1 could globally down-regulate translation by suppressing mTORC1 activity and activating GAAC pathway.

### LAT1 is involved in migration, invasion and tubular network formation of endothelial cells in angiogenic cellular processes

**Fig. 6:**
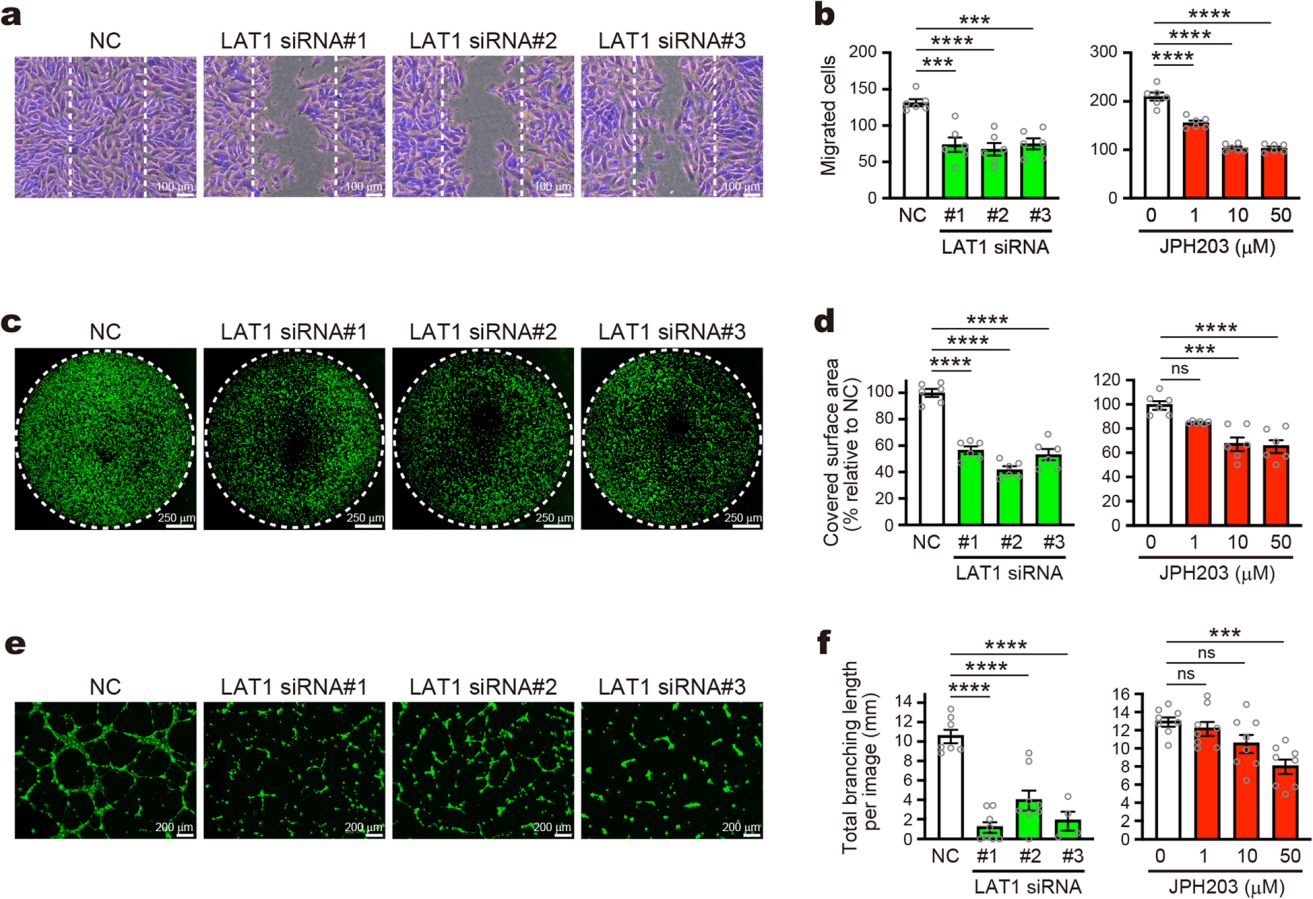
Suppression of migration, invasion and tube formation of endothelial cells by LAT1 knockdown and inhibition. **a** Wound healing assay using HUVECs transfected with control (*NC*) or LAT1-targeting siRNAs (*LAT1 siRNA #1–3*). **b** Quantification of migrated cell numbers in assays using LAT1 KD cells (*left*) and cells treated with JPH203 (0, 1, 10, 50 μM; *right*). **c** Invasion assay using LAT1 KD cells. **d** Quantification of covered surface areas in assays using LAT1 KD cells (*left*) and JPH203-treated cells (*right*). **e** Tube formation assay using LAT1 KD cells. **f** Quantification of total branching length of tubular network in assays using LAT1 KD cells (*left*), and JPH203-treated cells (*right*). **b**, **d**, *n* = 6; **f**, *n* = 7/8/8/4 for LAT1 KD, *n* = 8 for JPH203

Angiogenesis involves multiple cellular processes such as proliferation, migration, invasion, morphological change, and differentiation of endothelial cells. Because we confirmed LAT1 is essential for the proliferation of HUVECs (Fig. 5 and Supplementary Figure 5C), we further examined whether LAT1 is also involved in the other angiogenic cellular processes of HUVECs. In wound healing assay, LAT1 KD suppressed the migration (Fig. 6a), where the number of migrated cells was reduced to 50 ~ 60% of the control (Fig. 6b). LAT1 inhibitors, JPH203 and BCH, also suppressed the cell migration in concentration dependent manners (Fig. 6c and Supplementary Figure 5D). In the transwell invasion assay, LAT1 KD as well as LAT1 inhibitors reduced the number of cells migrated through a Matrigel layer (Fig. 6c, d and Supplementary Figure 5E). In the tube formation assay, LAT1 KD strongly disturbed the formation of tubular networks (Fig. 6e and f). Treatment with JPH203 also exhibited a reduction of tube formation. Effects of BCH, in which high concentration is required due to its lower affinity, were not evaluated because the tube formation was highly sensitive to the osmolality of culture medium. These results indicate that endothelial LAT1 contributes not only to proliferation but also to multiple angiogenic cellular processes, including migration, invasion, and tubular network formation.

### LAT1-mediated amino acid transport is indispensable for VEGF-A-dependent activation of mTORC1

In the signaling pathways regulating angiogenesis, VEGF-A and its cognate receptor VEGFR2 are known to play a central role [46, 47]. In our ex*-* and in vivo assays, VEGF-A was utilized as an angiogenic stimulant (Figs. 2 and 3). We also revealed that LAT1 in HUVECs is essential for angiogenic processes induced by VEGF-A stimulation (Fig. 6). We thus examined the contribution of LAT1 to VEGF-A-mediated pro-angiogenic intracellular signaling pathways under the condition comparable to that of in vitro assays shown in Fig. 6, i.e., HUVECs were starved for serum and growth factors (VEGF-A, FGF-2, EGF, and IGF-1), and then stimulated by VEGF-A.

**Fig. 7:**
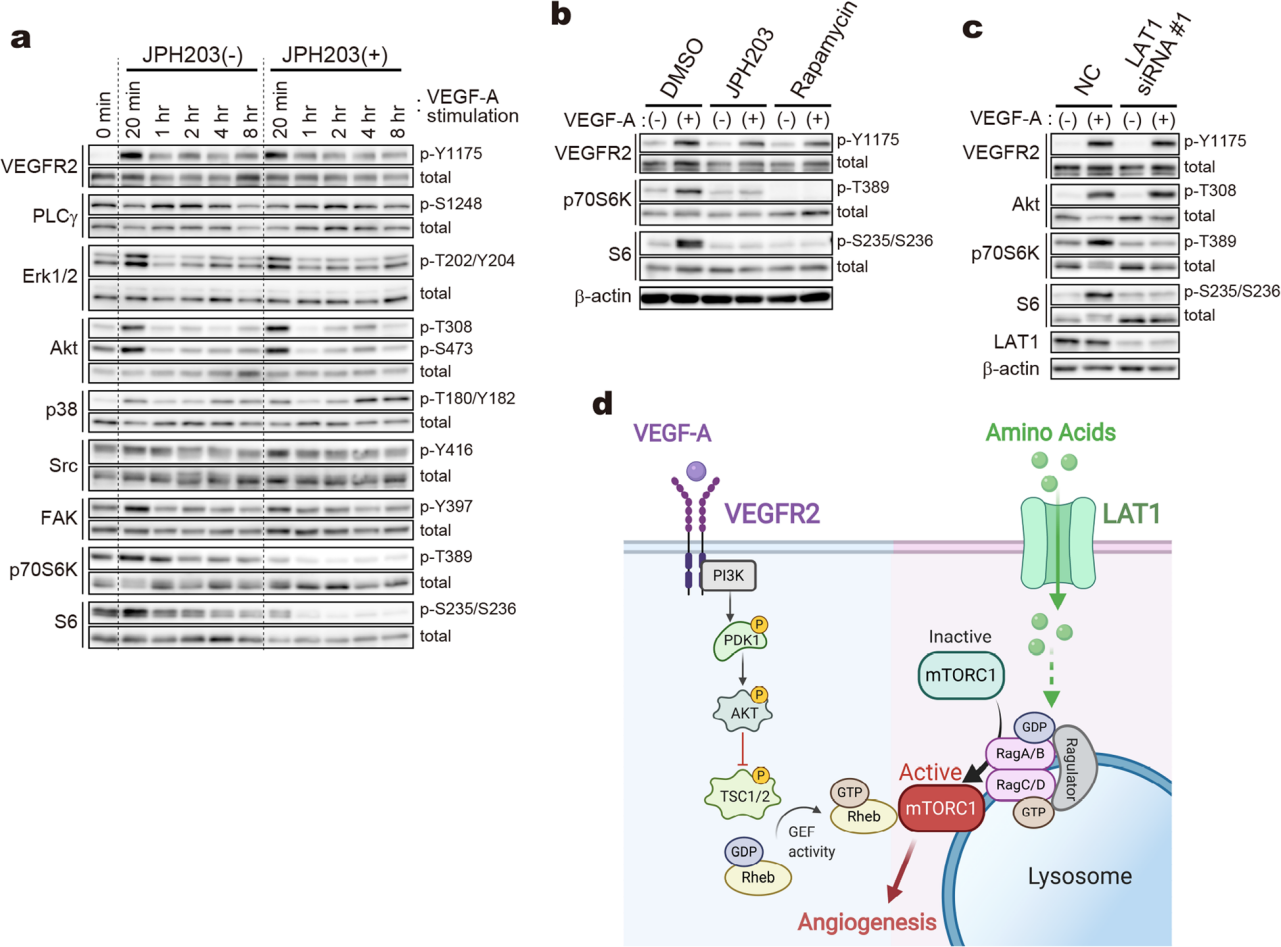
Effects of LAT1 knockdown and inhibition on VEGF-A-dependent signaling pathways. **a** HUVECs starved for serum and growth factors were stimulated with VEGF-A (10 ng/mL) in the presence or absence of JPH203 (50 μM). **b** and **c** Effects of JPH203 and rapamycin, and LAT1 KD on mTORC1- and GAAC pathways under stimulation with VEGF-A. HUVECs starved for serum and growth factors were stimulated with VEGF-A (10 ng/mL) for 20 min in the presence or absence of JPH203 (50 μM) or rapamycin (10 nM) (**b**). HUVECs transfected with control siRNA (*NC*) or LAT1-targeting siRNA (*LAT1 siRNA #1*) were starved and stimulated with VEGF-A (10 ng/mL) for 20 min (**c**). **d** Proposed molecular mechanism for the essential role of endothelial LAT1 in the VEGF-A-dependent activation of mTORC1. The LAT1-mediated amino acid signaling is independent of the PI3K-Akt axis in the downstream of VEGFR2, and is possibly mediated by Ragulator-Rag GTPase heterodimer complex that recruits inactive mTORC1 onto lysosomal surface for its interaction with kinase activator Rheb. The input of amino acid signaling is indispensable for the pro-angiogenic VEGF-A signaling to induce activation mTORC1 and subsequent angiogenesis

As shown in Fig. 7a, stimulation of the starved HUVECs with VEGF-A resulted in a transient increase of VEGFR2 phosphorylation at 20 min. The phosphorylation decreased with longer incubation time (> 1 h), but was sustained at a higher level than that before the treatment. Major downstream factors of VEGF-A/VEGFR2, including Erk1/2, Akt, p38, Src, FAK, p70S6K, and S6 ribosomal protein, also exhibited similar transient time courses in their phosphorylation, except PLCγ that showed a relatively delayed response. Treatment with JPH203 did not influence the phosphorylation of VEGFR2 and the downstream factors except for p70S6K and S6. The phosphorylation of p70S6K and S6 was drastically suppressed by JPH203 as early as 20 min after the stimulation, revealing that the VEGF-A-induced activation of mTORC1 is highly dependent on LAT1. It is especially of note that the phosphorylation of Akt at Thr308, locating in the upstream of mTORC1 [43], was less affected by JPH203. The decreased mTORC1 activity is, thus, most likely due to the reduced input of amino acid signaling mediated by Ragulator-Rag complex, which recruits mTORC1 onto lysosomal surface and facilitates its interaction with kinase activator Rheb in a manner generally independent of RTK-PI3K-Akt axis [43].

The inhibitory effects of JPH203 on VEGF-A-dependent mTORC1 activation were comparable to that of mTORC1 inhibitor rapamycin (Fig. 7b). Even though a residual phosphorylation of p70S6K was detected in JPH203-treated cells, the increase of phosphorylation in response to VEGF-A stimulation was mostly abolished. Furthermore, the phosphorylation of ribosomal S6 protein, the downstream of p70S6K, was suppressed to a similar extent by JPH203 and rapamycin. As shown in Fig. 7c, LAT1 KD also impaired the VEGF-A-dependent activation of p70S6K and S6, without affecting th`e phosphorylation levels of VEGFR2 and Akt (Thr308).

## Discussion

Our study revealed that amino acid transporter LAT1 expressed in tumor-associated endothelial cells is a novel key molecule in tumor angiogenesis. Extending previous studies with limited observations on a rat bladder carcinoma model [29] and human glioma tissues [30], we established the upregulation of LAT1 expression as a general characteristic of tumor-associated endothelial cells. The functional relevance of endothelial LAT1 to tumor angiogenesis was demonstrated in in vivo models by genetic and pharmacological inhibition of LAT1 (Fig. 4). Even though our study do not completely exclude a possibility that endothelial LAT1 also contributes to angiogenesis in certain physiological contexts, the endothelial cell-specific knockout of LAT1 in mouse strongly indicate that endothelial LAT1 is, at least, not essentially required for angiogenesis related to growth and survival. Furthermore, although LAT1 is expressed in brain epithelial cells as shown in Supplementary Figure 4 and in previous study [41], no obvious neurological adverse effects of JPH203 have been reported in previous studies using animal models [21, 24–27] and the first clinical trial [28]. We also did not observe any apparent neurological symptoms of mice in the present study. One possible explanation is that the inhibition of LAT1 can be compensated by the function of other amino acid transporters at blood brain barrier, the substrate specificity of which is overlapped with LAT1 [48]. Therefore, LAT1 seems to be a novel promising target in anti-angiogenic therapy. A strong anti-proliferative effect supported by a global down-regulation of translation could be achieved by endothelial LAT1 inhibition, not only by blocking the supply of amino acids as building blocks for protein synthesis, but also by interfering with amino acid signaling that regulates the initiation of translation (Fig. 5g and h). Such predominant inhibitory effects on translation are specific to LAT1 inhibition, clearly differentiating the mechanisms of action of LAT1 inhibitors from that of existing anti-angiogenic agents.

Intrinsic and acquired resistances against anti-angiogenic therapy often limit the benefits for patients [1, 2]. The multiple redundant and compensatory pro-angiogenic signaling pathways present in endothelial cells are supposed to play a crucial role in the resistance. A promising strategy to overcome the resistance would be to target multiple signaling pathways simultaneously. Accordingly, combination of FGFR inhibitor and bevacizumab in mouse tumor models almost completely suppressed tumor growth [49]. In pancreatic islet mouse tumors, resistance to VEGFR2 inhibitor was successfully impaired by the soluble decoy FGF receptor [6]. In this study, we demonstrated that LAT1 is indispensable for VEGF-A-dependent activation of mTORC1 (Fig. 7), which plays key roles in the cellular processes such as migration and tube formation in vitro as well as in in vivo angiogenesis [50–53]. Our results suggest that the roles of LAT1 in the activation of mTORC1 is mediated by Ragulator-Rag complex that is independent of RTK-PI3K-Akt axis. The amino acid signaling mediated by LAT1 seems to behave as a “gate-control” signal to permit the passage of pro-angiogenic VEGF-A signaling through mTORC1 to its downstream (Fig. 7d). Similar to the VEGFR signaling, multiple other pro-angiogenic RTKs including FGFR and TIE-2 share the PI3K-Akt axis that activates mTORC1 [46]. Therefore, the therapeutic inhibition of LAT1 with JPH203 could simultaneously interfere with not only VEGF-A/VEGFR2 signaling but also other pro-angiogenic signaling pathways at mTORC1, offering a possibility to circumvent the resistance resulting from the compensatory function of pro-angiogenic growth factor signaling.

While LAT1 is well-known as a “tumor cell-type transporter” highly and broadly upregulated in tumor cells to support their growth and proliferation, our study indicates a new insight into the dual functioning of LAT1 in tumor progression both in tumor cells and stromal endothelium. In this regard, we also would like to emphasize the unique dual mechanisms of action of LAT1 inhibitor JPH203 as anti-tumor agents, i.e. the well-established direct anti-proliferative effects on tumor cells through the inhibition of LAT1 in tumor cells and the anti-angiogenic effect through the inhibition of endothelial LAT1. A tempting speculation is that, when combined with other anti-angiogenic agents, administration of LAT1 inhibitors would suppress the compensatory paracrine secretion of pro-angiogenic factors from tumor cells, through the down-regulation of protein synthesis in tumor cells. Therefore, combinational therapies of LAT1 inhibitors with anti-angiogenic agents may show beneficial synergic anti-tumor effects with a lower risk of developing resistance.

Several lines of evidence indicate that tumor-associated endothelial cells are distinct from their normal counterparts in the expression of characteristic proteins [54–56]. Our present study indicates the increased LAT1 expression is also a part of such tumor endothelium-specific characteristics. We detected the endothelial LAT1 expression not only in tumor tissues but also in in vitro HUVEC cultures and in the endothelial cells from ex/in vivo angiogenesis assays, in which VEGF-A and FGF-2 were supplemented to culture media (Figs. 2, 3, and 5). These pro-angiogenic factors induced the expression of LAT1 in HUVECs at both mRNA and protein levels (Fig. 5a-c). It was previously reported that LAT1 is a direct target gene of oncogenic c-Myc [57, 58]. In the ontogenetic development, the expression of c-Myc in endothelial cells is regulated by VEGFR2 [59] and FGFR [60]. Even though further studies are awaited to elucidate the details, the tumor microenvironment rich in VEGF-A and FGF-2 may partly account for the upregulation of LAT1 in tumor-associated endothelium.

## Conclusion

In summary, we demonstrate that an amino acid transporter LAT1 is upregulated in tumor endothelium and plays fundamental roles in tumor angiogenesis. We revealed a cross-talk between LAT1-mediated amino acid signaling and growth factor-dependent pro-angiogenic signaling, converging on nutrient-sensing hub kinase mTORC1 to regulate angiogenesis. LAT1-targeting therapy may offer an ideal option to potentiate current cancer treatments especially for anti-angiogenic therapies.

## Supplementary Information

**Additional file 1: Supplementary Figure 1.** Reactivity and species specificity of generated mouse LAT1 antibodies. (A and B) Detection of mouse LAT1 by western blotting. HEK293T cells transiently co-expressing HA-tagged mouse LAT1 and 4F2hc (*mLAT1-HA*) or non-transfected control cells (*Mock*) were analyzed with mLAT1(C) antibody (A), and mLAT1(R) antibody (B). Membranes were reprobed with HA antibody. (C and D) Immunofluorescence in HEK293T cells transiently co-expressing HA-tagged mouse LAT1 and 4F2hc. Cells were stained with HA antibody and mLAT1(C) antibody (C) or mLAT1(R) antibody (D). (E and F) Species specificity of mouse LAT1 antibodies analyzed by western blotting. HT-29 cells and B16-F10 cells were analyzed with mLAT1(C) antibody (E), and mLAT1(R) antibody (F). Membranes were reprobed with LAT1 antibody (KE026, TransGenic), that recognizes both human and mouse LAT1. (G and H) Species specificity of mouse LAT1 antibodies analyzed by immunofluorescence. A549 cells and B16-F10 cells were stained with mLAT1(C) antibody and LAT1 antibody (G), or mLAT1(R) antibody and 4F2hc antibody (H). Nuclei stained with DAPI are shown in the merged images. **Supplementary Figure 2.** Generation of an exon 3-floxed conditional knockout allele of mouse LAT1 gene. Schematic genomic structures of alleles. Following elements are shown: exons (*purple boxes*), loxP sites (*yellow triangles*), FRT sites (*green triangles*), neomycin resistance gene cassettes (NEO, *gray boxes*), diphtheria toxin A-fragment (DTA, *pink box*), primers for genotyping PCR to distinguish floxed- and deleted alleles (*light blue triangles*), and probes for Southern blotting: 5′ probe (*red line*), 3′ probe (*red line*), and Neo probe (*purple line*). Dashed lines indicate long (5.4 kb) and short (2.3 kb) arms. **Supplementary Figure 3.** Effects of exon 3 deletion on l-[^14^C]leucine transport and expression of LAT1. (A) l-[^14^C]Leucine uptake of wild type LAT1 (*LAT1 WT*) and exon 3-deleted LAT1 (*LAT1-Δex3*) expressed alone or co-expressed with 4F2hc in *X. laevis* oocytes. Uptake was measured in Na^+^-free uptake buffer containing 100 μM l-[^14^C]leucine (3.3 Ci/mol) for 15 min. The data are shown as mean ± s.e.m. (*n* = 10/10/10/8/10/8 oocytes). Statistical analysis was conducted by one-way ANOVA followed by Tukey’s post-test. ****p < 0.0001; ns, not significant. (B) Expression of wild type LAT1 and exon 3-deleted LAT1 in *X. laevis* oocytes. Oocyte membrane fractions were analyzed by western blotting with mLAT1(R) antibody and 4F2hc antibody in the presence or absence of 100 mM DTT. Co-expression with 4F2hc decreased the amount of wild type- and exon 3-deleted LAT1, likely because of the competition for the capacity of oocyte’s translational machinery. The association between LAT1-Δex3 and 4F2hc was not detectable under no-reducing condition (*(−) DTT*), whereas wild type LAT1 was predominantly detected in the form of 4F2hc-LAT1 heterodimer (*arrows with red letters*). LAT1-Δex3 under non-reducing condition was predominantly detected as monomer or homodimer regardless of the presence of co-expressed 4F2hc. **Supplementary Figure 4.** Expression of LAT1 in endothelial cells of normal mouse tissues. Immunofluorescence of mouse LAT1 and CD34 in paraffin sections from BALB/c-nu/nu mouse tissues. Nuclei were stained with DAPI (*blue*, in merged images). LAT1 protein was detected in the endothelial cells using mouse-specific anti-LAT1 antibody (*LAT1(Ms)*), mLAT1(R) antibody. **Supplementary Figure 5**. Inhibition of LAT1 by BCH exhibits anti-angiogenic effect in in vitro, ex*/*in vivo angiogenesis assays and suppresses endothelial cell proliferation. (A) Aortic ring assay performed in the presence or the absence of 40 mM BCH. *Bar graph*; quantification of endothelial sprouts. (B) Fluorescent images of Matrigel plugs implanted with or without 40 mM BCH. *Bar graph*; quantification of FITC fluorescence. (C) Cell proliferation of HUVECs treated with BCH. The data are shown as mean ± s.d (*n* = 8). (D) Effects of BCH on cell migration of HUVECs. Quantification of migrated cell numbers in the wound healing assays using cells treated with BCH (0, 5, 10, 20 mM). (E) Effects of BCH on cell invasion. Quantification of covered surface areas in transwell invasion assays using cells treated with BCH. (A), *n* = 12; (B), *n* = 16; (D), (E), *n* = 6. The data are shown as mean ± s.e.m. Statistical analysis was conducted by unpaired two-tailed Student’s t-test for Fig. S4A and S4B, one-way ANOVA followed by Tukey’s post-test for Fig. S4D and S4E, and two-way ANOVA followed by Tukey’s post-test for Fig. S4C. * *p* < 0.05, ** *p* < 0.01, *** *p* < 0.001, **** *p* < 0.0001, ns, not significant. **Supplementary Figure 6.** Confirmation of gene disruption in conditional LAT1 knockout mice. (A) Genotyping of DOX-induced conditional LAT1 knockout mice. Genomic DNA was extracted from Matrigel-embedded aortic rings of *Lat1*^fl/fl^/*rtTA3*/*TetO-Cre* mice and control littermates (*Lat1*^fl/fl^/*TetO-Cre* or *Lat1*^fl/fl^/*rtTA3*). Aortic rings were cultured for 5 days in the presence or the absence of DOX. (B) Genotyping of endothelial cell-specific LAT1 knockout mice. Genomic DNA was extracted from 5-day-cultured Matrigel-embedded aortic rings of endothelial cell-specific LAT1 knockout mice (*Lat1*^fl/fl^*/Tek-Cre)* and its control *Lat1*^fl/fl^ littermates. (C) Immunofluorescence of LAT1 in brain sections of *Lat1*^fl/fl^*/Tek-Cre* and *Lat1*^fl/fl^ mice. LAT1 was stained with mLAT1(R) antibody. Blood vessels were labeled with intravenously injected FITC-Dextran. Nuclei were stained with DAPI (*blue*, in merged images).

## Data Availability

All data generated or analysed during this study are included in this published article [and its supplementary information files].

## Abbreviations

LAT1: L-type amino acid transporter 1
4F2hc: 4F2 heavy chain
PDA: Pancreatic ductal adenocarcinoma
VEGF-A: Vascular endothelial growth factor A-165
FGF-2: Fibroblast growth factor-2
EGF: Epidermal growth factor
IGF-1: Insulin-like growth factor 1
RTK: Receptor tyrosine kinase
BCH: 2-Aminobicyclo[2.2.1]heptane-2-carboxylic acid
JPH203: (S)-2-amino-3-(4-((5-amino-2-phenylbenzo[d]oxazol-7-yl)methoxy)-3,5-dichlorophenyl) propanoic acid
SBECD: Sulfobutylether-β-cyclodextrin
HUVECs: Human umbilical vein endothelial cells
mTORC1: Mechanistic target of rapamycin complex 1
GAAC: General amino acid control
Gcn2: General control nonderepressible 2
VEGFR2: Vascular endothelial growth factor receptor kinase 2
FGFR: Fibroblast growth factor receptor
PI3K: Phosphoinositide 3-kinase
Akt: Protein kinase B
FAK: Focal adhesion kinase
Erk: Extracellular signal-regulated kinase
PLC: Phospholipase C
eIF2: Eukaryotic initiation factor 2
PFA: paraformaldehyde
DOX: doxycycline
