# Supplementary material for: Amino acid transporter LAT1 in tumor-associated vascular endothelium promotes angiogenesis by regulating cell proliferation and VEGF-A-dependent mTORC1 activation
Source: J Exp Clin Cancer Res. 2020 Nov 30;39:266. doi: 10.1186/s13046-020-01762-0 (PMC7702703; doi:10.1186/s13046-020-01762-0)
Supplement: Supplementary file 1 — Additional file 1: Supplementary Figure 1. Reactivity and species specificity of generated mouse LAT1 antibodies. (A and B) Detection of mouse LAT1 by western blotting. HEK293T cells transiently co-expressing HA-tagged mouse LAT1 and 4F2hc (mLAT1-HA) or non-transfected control cells (Mock) were analyzed with mLAT1(C) antibody (A), and mLAT1(R) antibody (B). Membranes were reprobed with HA antibody. (C and D) Immunofluorescence in HEK293T cells transiently co-expressing HA-tagged mouse LAT1 and 4F2hc. Cells were stained with HA antibody and mLAT1(C) antibody (C) or mLAT1(R) antibody (D). (E and F) Species specificity of mouse LAT1 antibodies analyzed by western blotting. HT-29 cells and B16-F10 cells were analyzed with mLAT1(C) antibody (E), and mLAT1(R) antibody (F). Membranes were reprobed with LAT1 antibody (KE026, TransGenic), that recognizes both human and mouse LAT1. (G and H) Species specificity of mouse LAT1 antibodies analyzed by immunofluorescence. A549 cells and B16-F10 cells were stained with mLAT1(C) antibody and LAT1 antibody (G), or mLAT1(R) antibody and 4F2hc antibody (H). Nuclei stained with DAPI are shown in the merged images. Supplementary Figure 2. Generation of an exon 3-floxed conditional knockout allele of mouse LAT1 gene. Schematic genomic structures of alleles. Following elements are shown: exons (purple boxes), loxP sites (yellow triangles), FRT sites (green triangles), neomycin resistance gene cassettes (NEO, gray boxes), diphtheria toxin A-fragment (DTA, pink box), primers for genotyping PCR to distinguish floxed- and deleted alleles (light blue triangles), and probes for Southern blotting: 5′ probe (red line), 3′ probe (red line), and Neo probe (purple line). Dashed lines indicate long (5.4 kb) and short (2.3 kb) arms. Supplementary Figure 3. Effects of exon 3 deletion on l-[14C]leucine transport and expression of LAT1. (A) l-[14C]Leucine uptake of wild type LAT1 (LAT1 WT) and exon 3-deleted LAT1 (LAT1-Δex3) expressed alone or co-expresse [file 13046_2020_1762_MOESM1_ESM.docx]

**Supplementary Figures**

Amino acid transporter LAT1 in tumor-associated vascular endothelium promotes angiogenesis by regulating cell proliferation and VEGF-A-dependent mTORC1 activation

Lili Quan^1#^, Ryuichi Ohgaki^1#^, Saori Hara^1^, Suguru Okuda^1^, Ling Wei^1, 2^, Hiroki Okanishi^1^, Shushi Nagamori^3^, Hitoshi Endou^4^, Yoshikatsu Kanai^1, 5^

# These authors contributed equally to this work.

Authors and affiliations

^1^ Department of Bio-system Pharmacology, Graduate School of Medicine, Osaka University, 2-2 Yamadaoka, Suita, 565-0871 Osaka, Japan.

^2^ Present address: School of Traditional Chinese Medicine, Guangdong Pharmaceutical University, Guangzhou, 510006 Guangdong, China.

^3^ Department of Laboratory Medicine, The Jikei University School of Medicine, Minato-ku, 634-8521 Tokyo, Japan.

^4^ J-Pharma Co., Ltd, Yokohama, 230-0046 Kanagawa, Japan.

^5^ Integrated Frontier Research for Medical Science Division, Institute for Open and Transdisciplinary Research Initiatives, Osaka University, Suita, 565-0871 Osaka, Japan.

**Supplementary Figure 1**


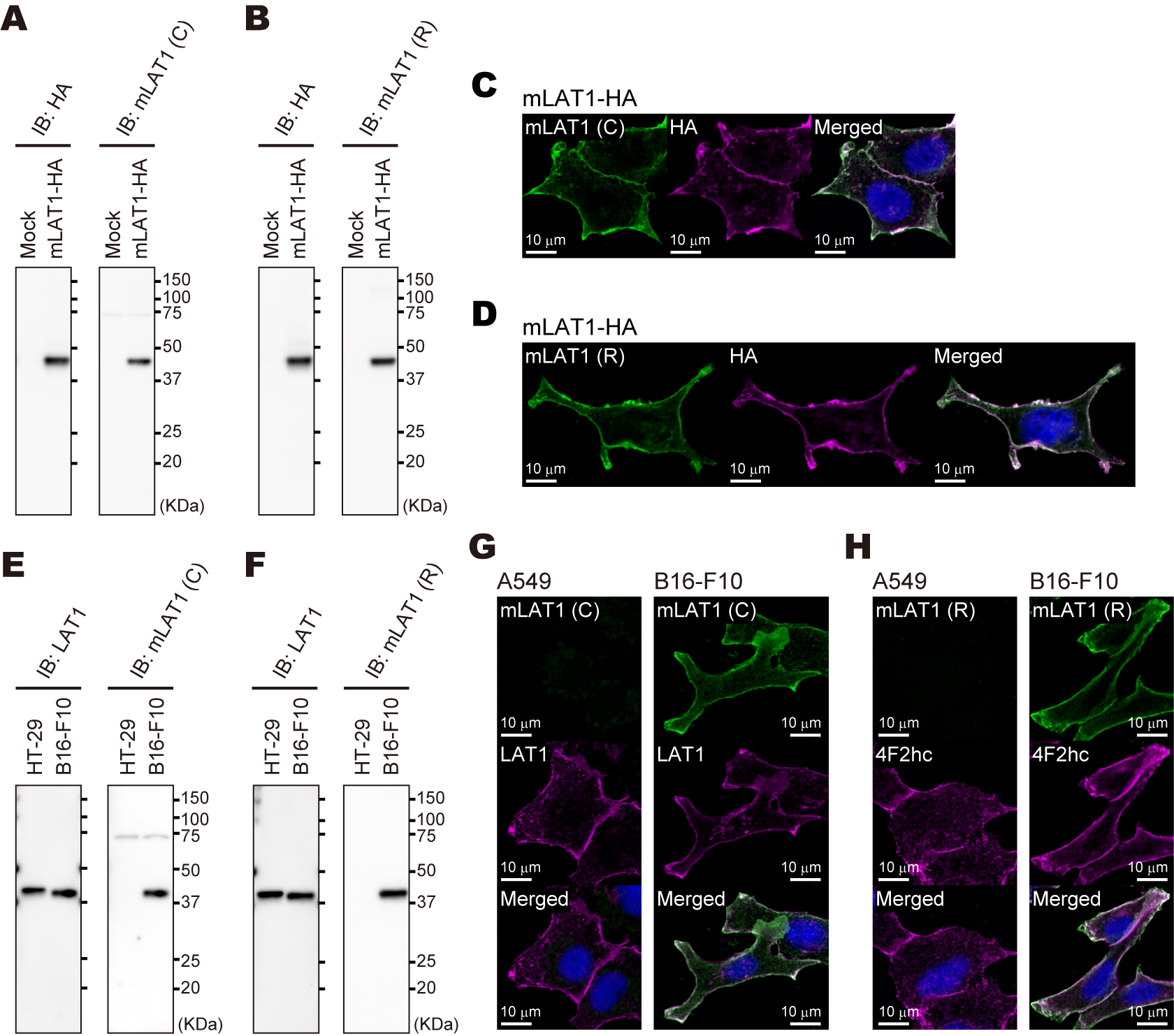


## **Supplementary Figure 1 Reactivity and species specificity of generated mouse LAT1 antibodies.** (**A** and **B**) Detection of mouse LAT1 by western blotting. HEK293T cells transiently co-expressing HA-tagged mouse LAT1 and 4F2hc (*mLAT1-HA*) or non-transfected control cells (*Mock*) were analyzed with mLAT1(C) antibody (**A**), and mLAT1(R) antibody (**B**). Membranes were reprobed with HA antibody. (**C** and **D**) Immunofluorescence in HEK293T cells transiently co-expressing HA-tagged mouse LAT1 and 4F2hc. Cells were stained with HA antibody and mLAT1(C) antibody (**C**) or mLAT1(R) antibody (**D**). (**E** and **F**) Species specificity of mouse LAT1 antibodies analyzed by western blotting. HT-29 cells and B16-F10 cells were analyzed with mLAT1(C) antibody (**E**), and mLAT1(R) antibody (**F**). Membranes were reprobed with LAT1 antibody (KE026, TransGenic), that recognizes both human and mouse LAT1. (**G** and **H**) Species specificity of mouse LAT1 antibodies analyzed by immunofluorescence. A549 cells and B16-F10 cells were stained with mLAT1(C) antibody and LAT1 antibody (**G**), or mLAT1(R) antibody and 4F2hc antibody (**H**). Nuclei stained with DAPI are shown in the merged images.

**
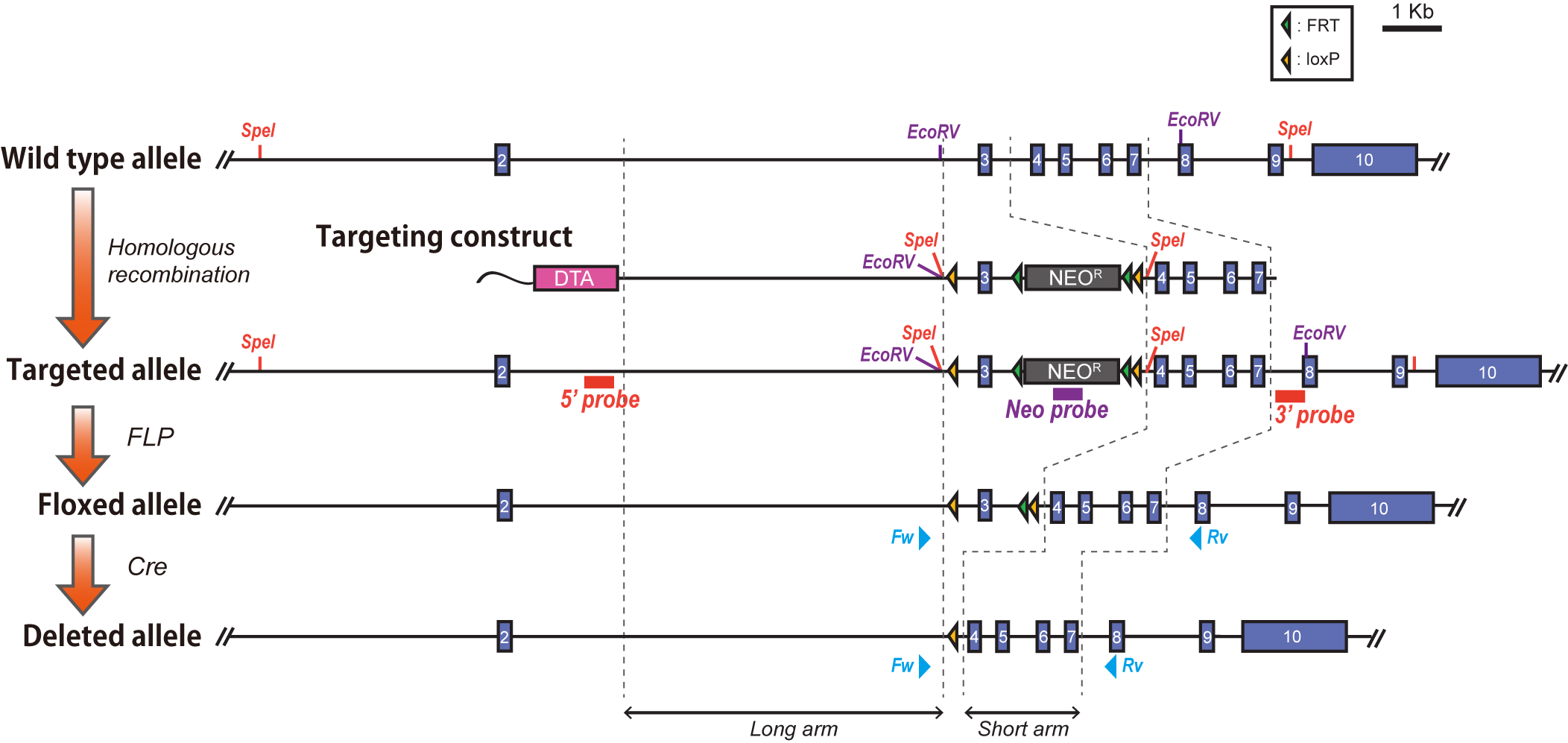
Supplementary Figure 2**

## **Supplementary Figure 2 Generation of an exon 3-floxed conditional knockout allele of mouse LAT1 gene.** Schematic genomic structures of alleles. Following elements are shown: exons (*purple boxes*), loxP sites (*yellow triangles*), FRT sites (*green triangles*), neomycin resistance gene cassettes (NEO, *gray boxes*), diphtheria toxin A-fragment (DTA, *pink box*), primers for genotyping PCR to distinguish floxed- and deleted alleles (*light blue triangles*), and probes for Southern blotting : 5’ probe (*red line*), 3’ probe (*red line*), and Neo probe (*purple line*). Dashed lines indicate long (5.4 kb) and short (2.3 kb) arms.

**
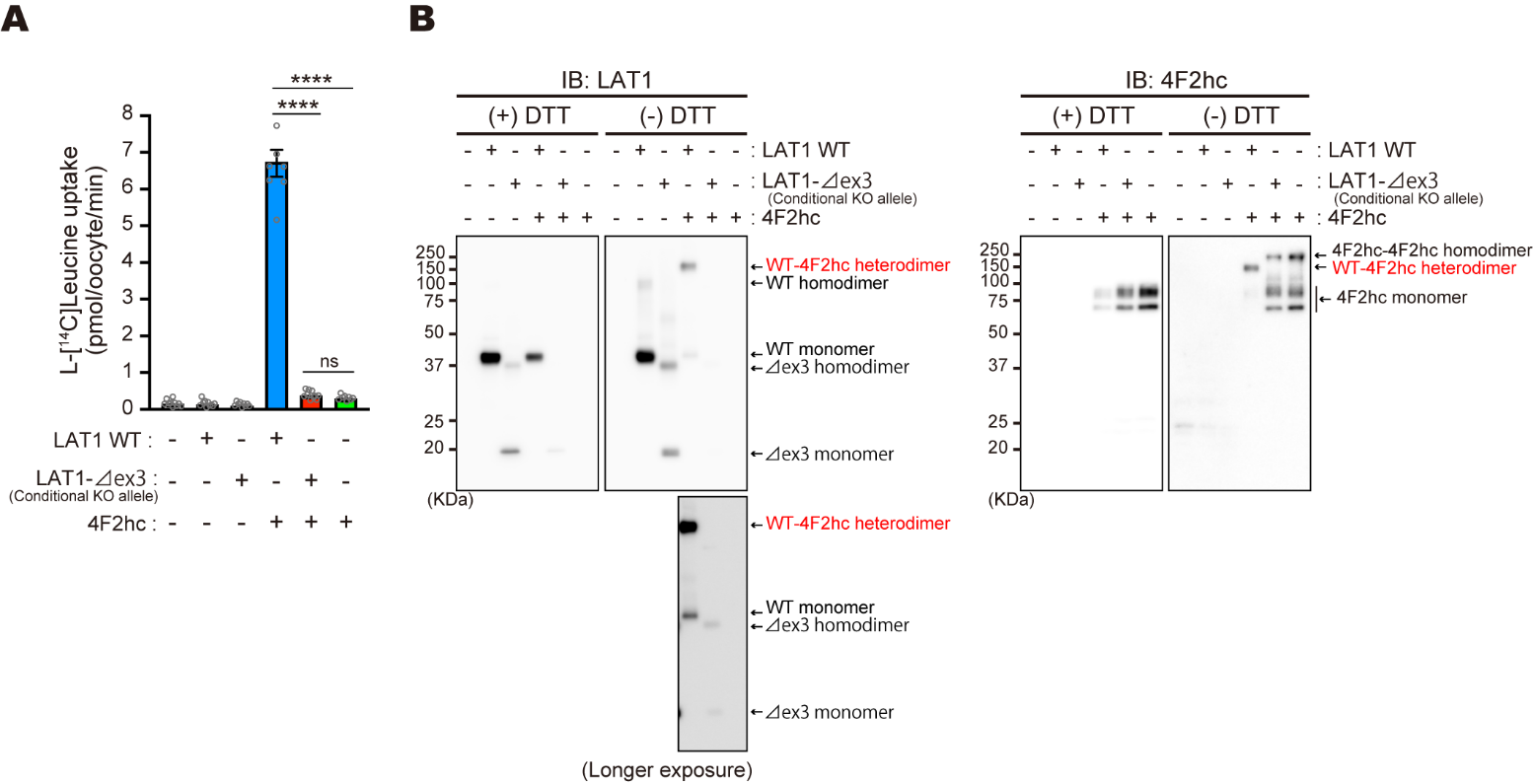
Supplementary Figure 3**

**Supplementary Figure 3 Effects of exon 3 deletion on l-[^14^C]leucine transport and expression of LAT1.** (**A**) l-[^14^C]Leucine uptake of wild type LAT1 (*LAT1 WT*) and exon 3-deleted LAT1 (*LAT1-Δex3*) expressed alone or co-expressed with 4F2hc in *X. laevis* oocytes. Uptake was measured in Na^+^-free uptake buffer containing 100 μM l-[^14^C]leucine (3.3 Ci/mol) for 15 min. The data are shown as mean ± s.e.m (n = 10/10/10/8/10/8 oocytes). Statistical analysis was conducted by one-way ANOVA followed by Tukey’s post-test. *****p* < 0.0001; ns, not significant. (**B**) Expression of wild type LAT1 and exon 3-deleted LAT1 in *X. laevis* oocytes. Oocyte membrane fractions were analyzed by western blotting with mLAT1(R) antibody and 4F2hc antibody in the presence or absence of 100 mM DTT. Co-expression with 4F2hc decreased the amount of wild type- and exon 3-deleted LAT1, likely because of the competition for the capacity of oocyte’s translational machinery. The association between LAT1-Δex3 and 4F2hc was not detectable under no-reducing condition (*(-) DTT*), whereas wild type LAT1 was predominantly detected in the form of 4F2hc-LAT1 heterodimer (*arrows with red letters*). LAT1-Δex3 under non-reducing condition was predominantly detected as monomer or homodimer regardless of the presence of co-expressed 4F2hc.

**Supplementary Figure 4**


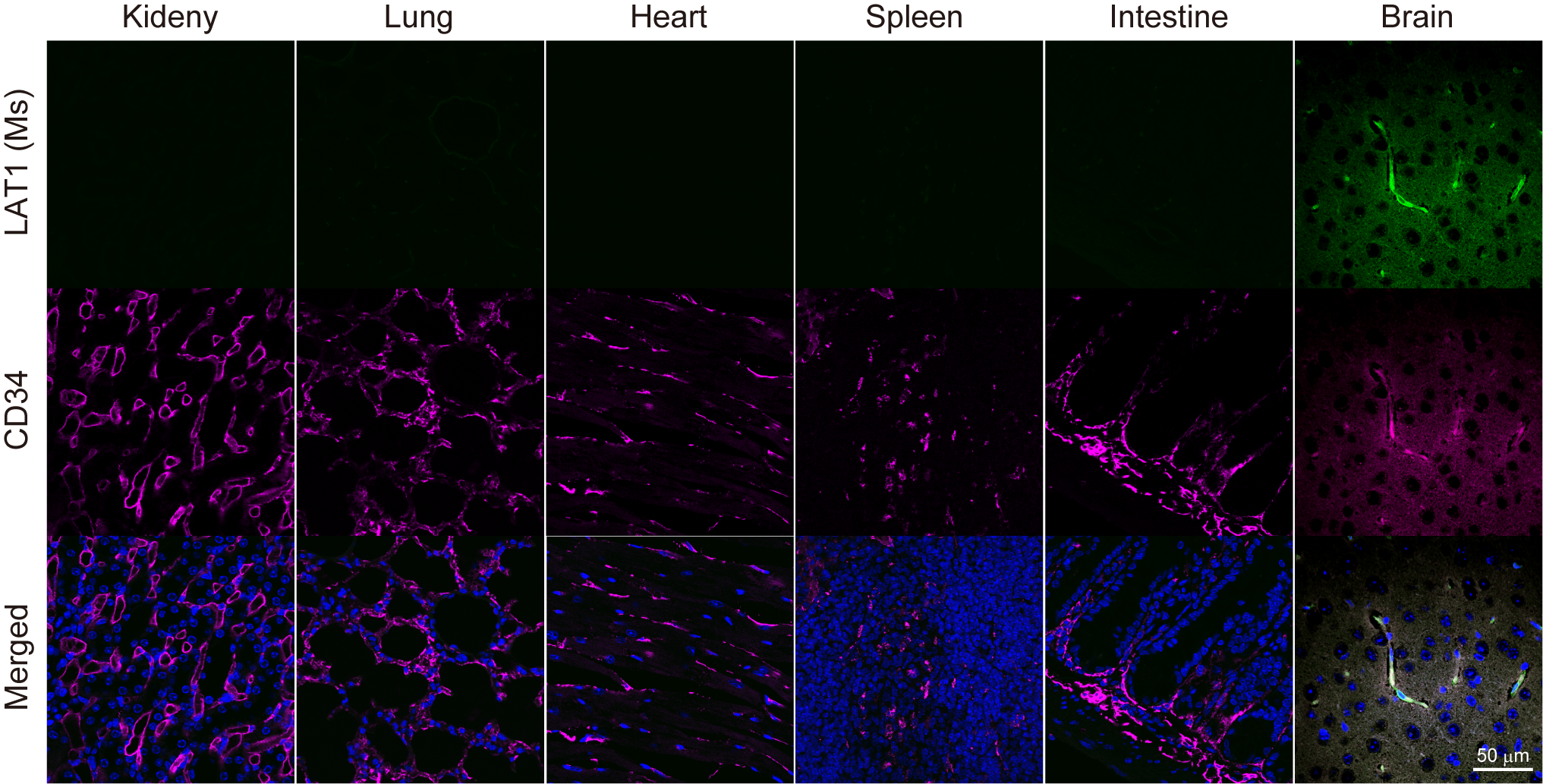


## **Supplementary Figure 4 Expression of LAT1 in endothelial cells of normal mouse tissues.** Immunofluorescence of mouse LAT1 and CD34 in paraffin sections from BALB/c-nu/nu mouse tissues. Nuclei were stained with DAPI (*blue*, in merged images). LAT1 protein was detected in the endothelial cells using mouse-specific anti-LAT1 antibody (*LAT1(Ms)*), mLAT1(R) antibody.

**Supplementary Figure 5**


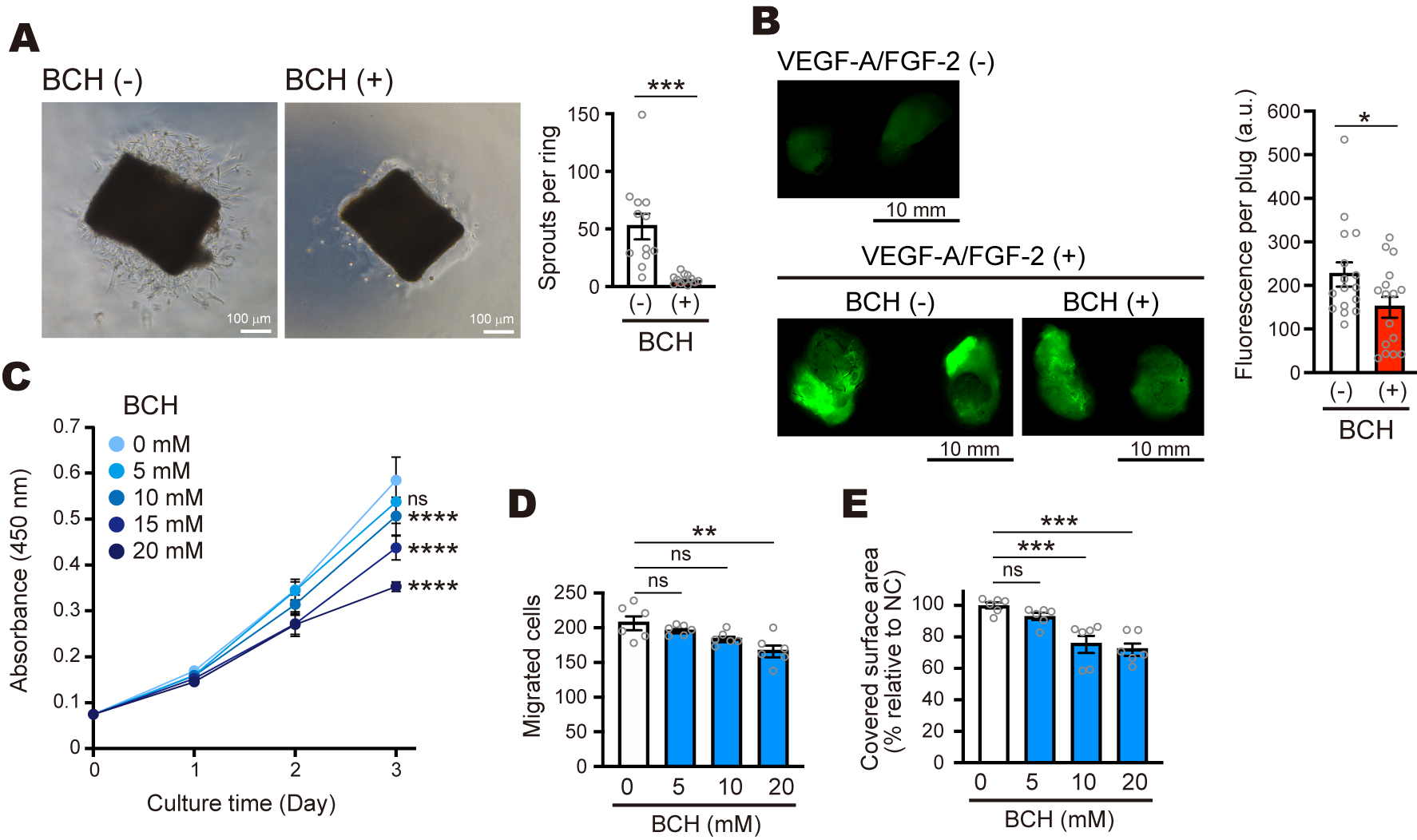


## **Supplementary Figure 5 Inhibition of LAT1 by BCH exhibits anti-angiogenic effect in *in vitro*, *ex/in* vivo angiogenesis assays and suppresses endothelial cell proliferation.** (**A**) Aortic ring assay performed in the presence or the absence of 40 mM BCH. *Bar graph*; quantification of endothelial sprouts. (**B**) Fluorescent images of Matrigel plugs implanted with or without 40 mM BCH. *Bar graph*; quantification of FITC fluorescence. (**C**) Cell proliferation of HUVECs treated with BCH. The data are shown as mean ± s.d (n = 8). (**D**) Effects of BCH on cell migration of HUVECs. Quantification of migrated cell numbers in the wound healing assays using cells treated with BCH (0, 5, 10, 20 mM). (**E**) Effects of BCH on cell invasion. Quantification of covered surface areas in transwell invasion assays using cells treated with BCH. (**A**), n = 12; (**B**), n = 16; (**D**), (**E**), n = 6. The data are shown as mean ± s.e.m. Statistical analysis was conducted by unpaired two-tailed Student’s t-test for Fig. S4A and S4B, one-way ANOVA followed by Tukey’s post-test for Fig. S4D and S4E, and two-way ANOVA followed by Tukey’s post-test for Fig. S4C. * *p* < 0.05, ** *p* < 0.01, *** *p* < 0.001, **** *p* < 0.0001, ns, not significant.

**Supplementary Figure 6**


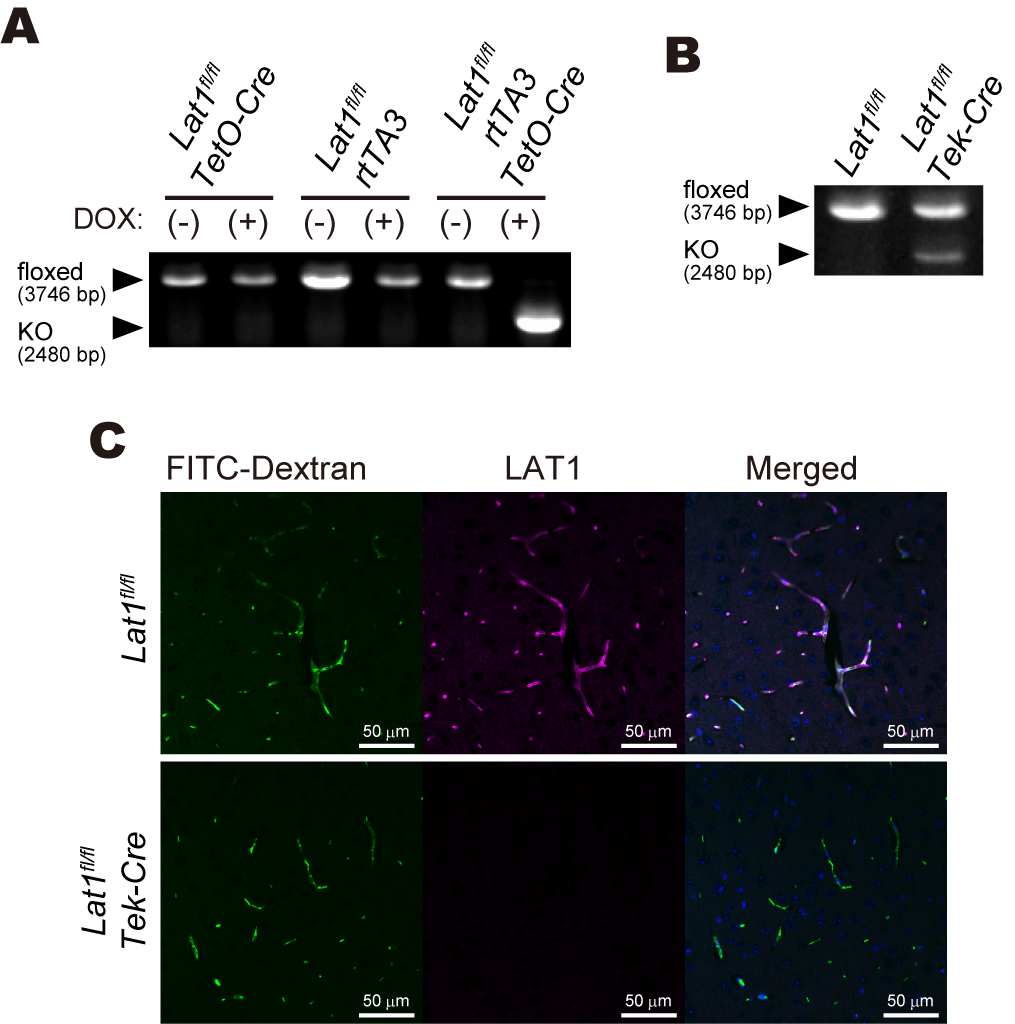


## **Supplementary Figure 6 Confirmation of gene disruption in conditional LAT1 knockout mice.** (**A**) Genotyping of DOX-induced conditional LAT1 knockout mice. Genomic DNA was extracted from Matrigel-embedded aortic rings of *Lat1*^fl/fl^/*rtTA3*/*TetO-Cre* mice and control littermates (*Lat1*^fl/fl^/*TetO-Cre* or *Lat1*^fl/fl^/*rtTA3*). Aortic rings were cultured for 5 days in the presence or the absence of DOX. (**B**) Genotyping of endothelial cell-specific LAT1 knockout mice. Genomic DNA was extracted from 5-day-cultured Matrigel-embedded aortic rings of endothelial cell-specific LAT1 knockout mice (*Lat1*^fl/fl^*/Tek-Cre)* and its control *Lat1*^fl/fl^ littermates. (**C**) Immunofluorescence of LAT1 in brain sections of *Lat1*^fl/fl^*/Tek-Cre* and *Lat1*^fl/fl^ mice. LAT1 was stained with mLAT1(R) antibody. Blood vessels were labeled with intravenously injected FITC-Dextran. Nuclei were stained with DAPI (*blue*, in merged images).
